# Supplementary material for: Label-free adaptive optics single-molecule localization microscopy for whole zebrafish
Source: Nat Commun. 2023 Jul 13;14:4185. doi: 10.1038/s41467-023-39896-2 (PMC10344925; doi:10.1038/s41467-023-39896-2)
Supplement: Supplementary file 1 — Supplementary Information [file 41467_2023_39896_MOESM1_ESM.pdf]

Supplementary Information

Label-free adaptive optics single-molecule localization microscopy for whole zebrafish

Sanghyeon Park[1,2,+], Yonghyeon Jo[1,2,+], Minsu Kang[3], Jin Hee Hong[1], Sangyoon Ko[3], Suhyun Kim[4], Sangjun Park[5,6], Hae-Chul Park[4], Sang-Hee Shim[3,*], and Wonshik Choi[1,2,*]

[1]*Center for Molecular Spectroscopy and Dynamics, Institute for Basic Science, Seoul, Republic of Korea (02841)*

[2]*Department of Physics, Korea University, Seoul, Republic of Korea (02841)*

[3]*Department of Chemistry, Korea University, Seoul, Republic of Korea (02841)*

[4]*Department of Biomedical Sciences, Korea University, Ansan, Republic of Korea (15355)*

[5]*Department of Medical Life Sciences, The Catholic University of Korea, Seoul, Republic of Korea (06591)*

[6]*Department of Biomedicine & Health Sciences, The Catholic University of Korea, Seoul, Republic of Korea (06591)*

[+]*These authors contributed equally to this work*

[*]*sangheeshim@korea.ac.kr and wonshik@korea.ac.kr*

Contents

Supplementary Figures and Table
- Supplementary Figure 1. 2
- Supplementary Figure 2. 3
- Supplementary Figure 3. 4
- Supplementary Figure 4. 5
- Supplementary Figure 5. 7
- Supplementary Figure 6 8
- Supplementary Table 1. 9

Supplementary Note 1 10
- Supplementary Figure 7. 11
- Supplementary Figure 8. 12
- Supplementary Figure 9. 13
- Supplementary Figure 10. 14
- Supplementary Figure 11. 15
- Supplementary Figure 12. 16
- Supplementary Figure 13. 18

Supplementary Note 2 19
- Supplementary Figure 14. 19
- Supplementary Figure 15. 20
- Supplementary Figure 16. 21
- Supplementary Figure 17. 22
- Supplementary Figure 18. 23

Supplementary References 24

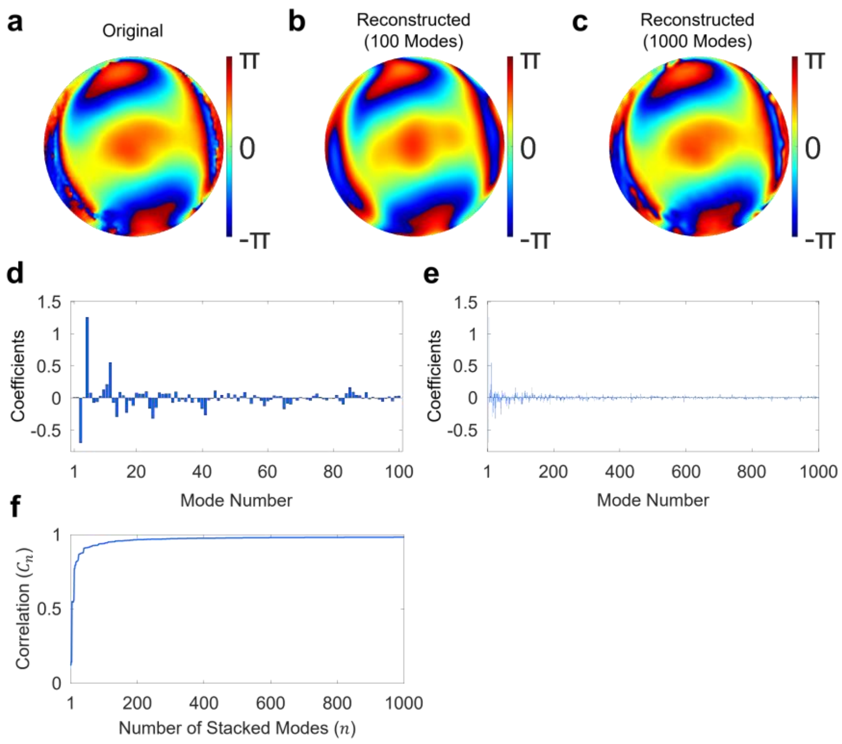

Supplementary Figure 1. Aberration map decomposition by Zernike modes. a Original aberration map identified by CLASS shown in the inset of Fig. 2c. **b** Reconstructed aberration map using the first 100 Zernike modes. It was quite similar to the original map in **a**, but slight differences were observed in the high spatial frequency region. Its correlation with the original aberration map was 0.941. **c** Same as **b**, but using the first 1000 Zernike modes. This aberration map was almost identical to the original one. Its correlation with the original aberration map was 0.985. **d**, **e** Coefficients of first 100 and 1000 Zernike modes constituting the reconstructed aberration maps in **b** and **c**, respectively. Note that the mode number over 20 still had non-negligible contributions. This indicates the complexity of the corrected aberration in this work considering that most of the previous reports dealt with aberrations composed of the first ~20 Zernike modes[1–4]. **f** Correlation C_n between the original aberration map and the aberration maps reconstructed using first n Zernike modes $(n = 1, 2, \cdots, 1000)$. Correlation C_n increases as the total number n of Zernike modes used for reconstruction increases. Correlations C_n for n = 20, 100, 1000 were C_{20} = 0.821, C_{100} = 0.941, and C_{1000} = 0.985, respectively.

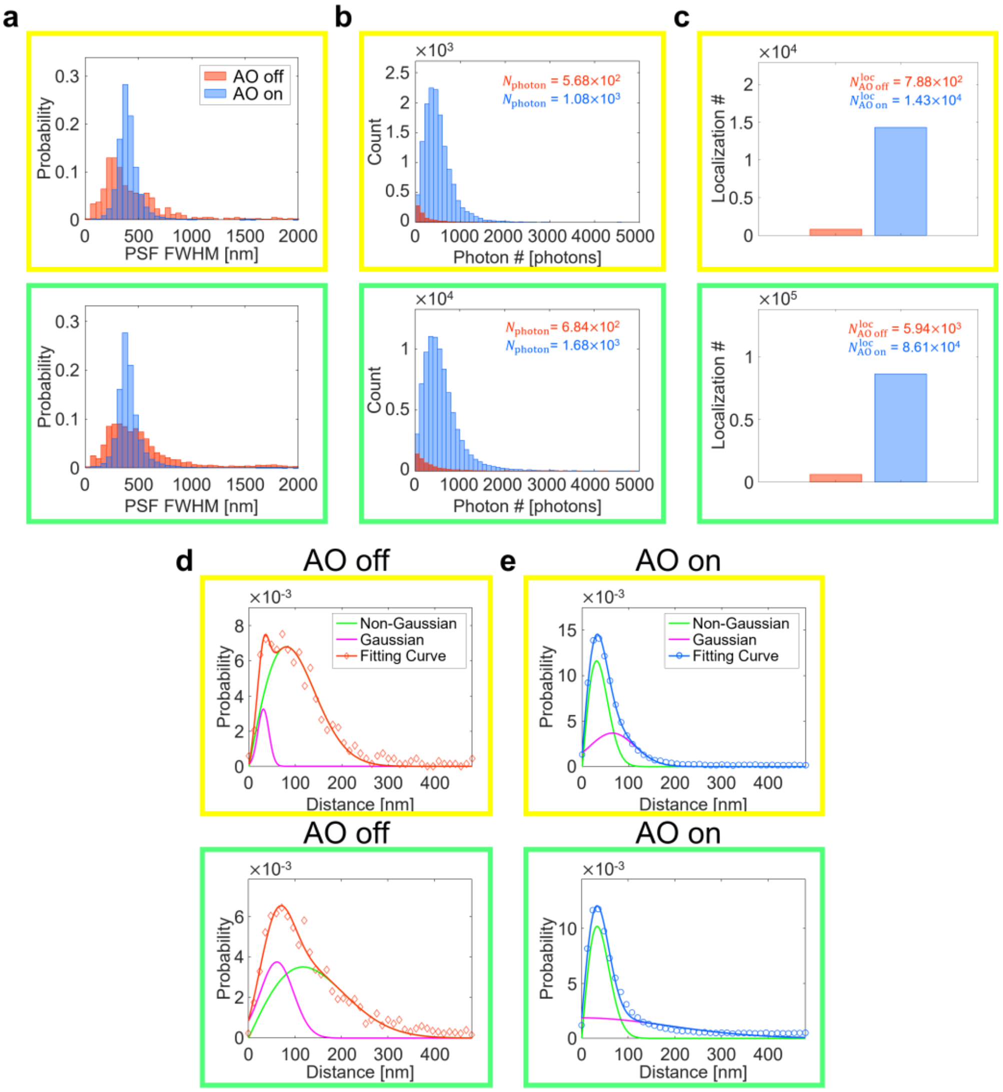

Supplementary Figure 2. Comparison of SMLM quality metrics in Figs. 2c, d. Four different regions where single microtubules were isolated were selected as sparse regions in Figs. 2c, d (including yellow boxes). Likewise, two different regions where microtubules were confluent were selected as dense regions in Figs. 2c, d (including green boxes). Then, several parameters were extracted from these two types of regions. **a** Probability distribution of PSF FWHMs. In both cases, it is noteworthy that PSF FWHMs were widely spread without AO, extending even beyond 1 µm. However, the distributions were much narrower with AO. This supports that the localization was robust with AO. **b** Histograms of photon number per emission PSF. **c** Bar graphs of the total number of localizations. There was a remarkable increase with AO. **d**, **e** Probability density curves obtained by the nearest neighbor analysis of SMLM images without (**d**) and with (**e**) AO as in Figs. 2j, l, but with separate fitting curves for a non-Gaussian and a Gaussian correction term (see "SMLM analysis" section in Methods for the mathematical expression). With AO, the non-Gaussian curves, accounting for individual single molecules, dominantly contributed to the probability density curve in both sparse (yellow boxes in Figs. 2c, d) and dense (green boxes in Figs. 2c, d) regions (**e**). This indicates that AO worked well regardless of the density of microtubules. Without AO, however, a different tendency was observed. In the dense region (green boxes in Figs. 2c, d), the Gaussian curve, describing the effect of different neighboring molecules, made a significant contribution. By comparing AO-off and -on raw images (Supplementary Video 1), it was visually confirmed that overlap among PSFs of neighboring molecules was pronounced without AO. This explains why most of microtubules were not recognizable in the dense region without AO (green box in Fig. 2g). In the sparse region (yellow boxes in Figs. 2c, d), the non-Gaussian curve was dominant in both cases of AO off and on (**d**). However, the FWHM of the non-Gaussian curve, i.e., localization precision, was much broader without AO (57 nm) than with AO (22 nm).

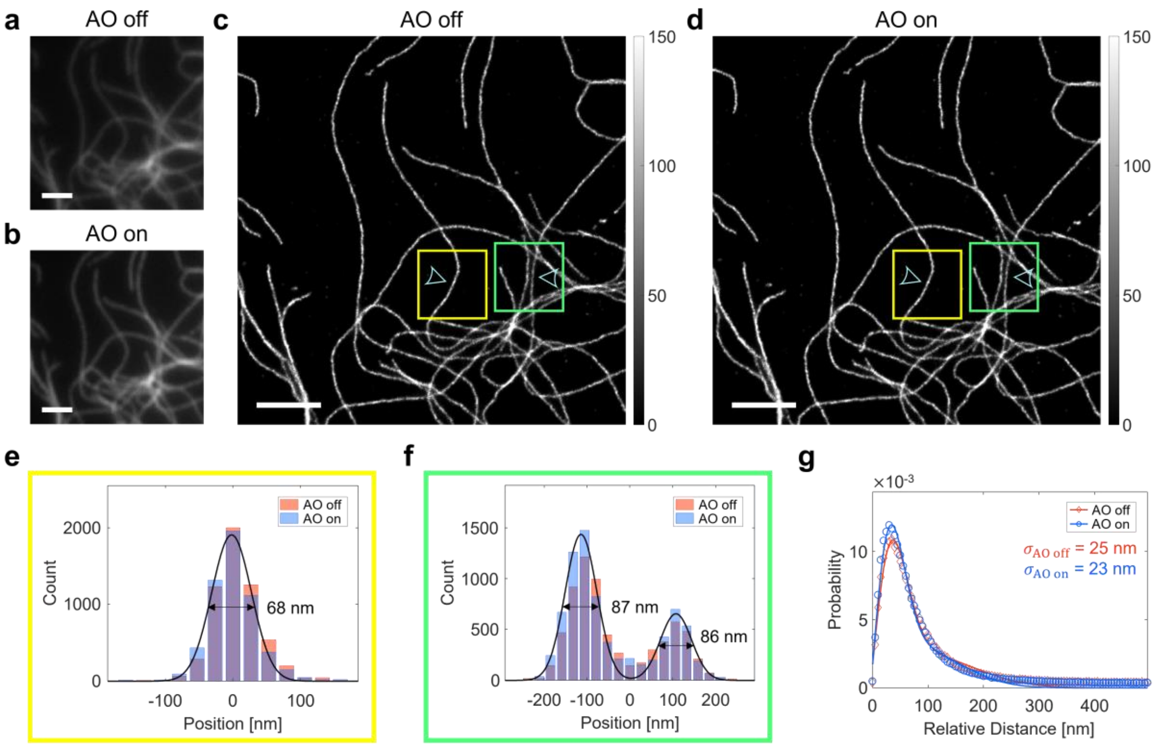

Supplementary Figure 3. SMLM images of cells without an aberration layer. a, **b** Diffraction-limited fluorescence images without (**a**) and with (**b**) AO, respectively. Images are normalized with respect to AO on. Scale bars indicate 2.5 μm. **c**, **d** SMLM images without (**c**) and with (**d**) AO, respectively. Here, only SMLM setup aberration was corrected when acquiring AO-on raw images of single-molecule blinking. Color bars indicate localization numbers. Scale bars indicate 2.5 μm. **e**, **f** Cross-sectional profiles at arrowheads in the yellow and green boxes in **c**, **d**, respectively. **g** Nearest neighbor analysis results.

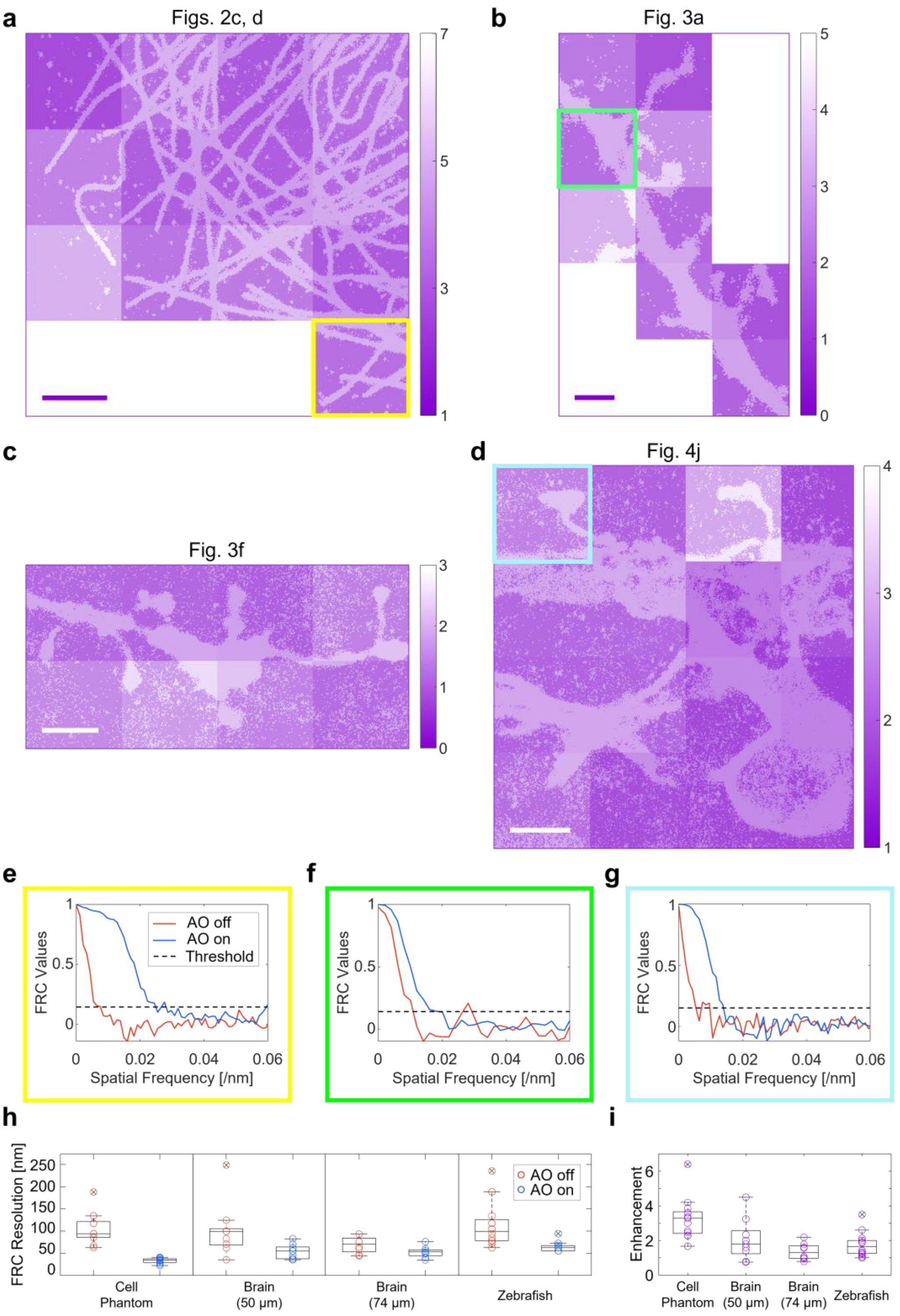

Supplementary Figure 4. Fourier ring correlation (FRC) analysis of SMLM images. a-d Enhancement of local FRC resolutions in Figs. 2c, 2d, 3a, 3f, and 4j, respectively. FRC resolutions were calculated as the reciprocals of cut-off spatial frequencies at which FRC value equals to 1/7[5]. Excluded areas are where no notable structures exist, which cause abnormally large values. Color bars indicate FRC resolution enhancements. Scale bars indicate 2.5 μm (**a**, **c**, **d**) and 2 μm (**b**). **e-g** Representative FRC spectra for the colored boxes indicated in **a**, **b**, and **d**, respectively. Nearest neighbor analysis curves in Figs. 3e, k showed mild distinctions between AO off and on though there were ~2-fold reductions in localization precisions with AO. However, FRC curve revealed an obvious broadening of FRC spectrum with AO. This is because FRC analysis incorporates the contribution of

localization number as well as that of localization precision. **h**, **i** Box plots showing AO-off and -on FRC resolutions (**h**) and FRC resolution enhancements (**i**) for n = 13 (cell phantom), 9 (brain 50 μm), 8 (brain 74 μm), and 16 (zebrafish) sub-areas in **a**-**d**. Central lines indicate medians. Bottom and top edges of the boxes indicate 25th and 75th percentiles, respectively. Whiskers extend from minima to maxima (except outliers marked with crosses). Note that AO-on FRC resolutions were well below 100 nm in all cases while many of AO-off resolutions were above 100 nm. This is consistent with the experimental observation that structures smaller than 100 nm were visible only with AO. Meanwhile, FRC resolution enhancements were largely similar in all local regions, suggesting that AO worked homogeneously everywhere.

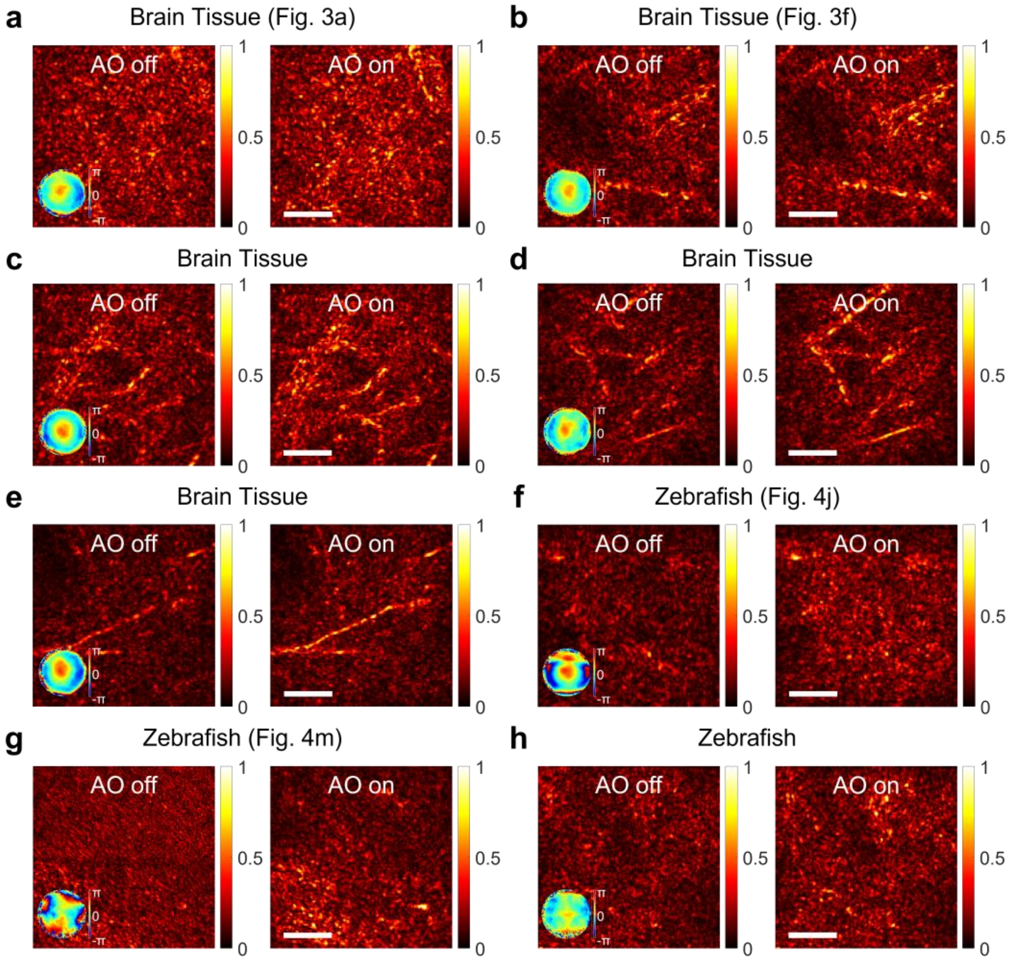

Supplementary Figure 5. Reflection images of various samples. a-**h** AO-off and -on reflectance images of various samples. Insets in each AO-off image indicate aberration correction maps. Color bars indicate normalized image intensity. Scale bars indicate 5 μm. In each sample, AO-off and -on images were normalized with AO-on image. Technically, AO-off images correspond to CASS images and AO-on images to aberration-corrected CASS images (see "Detailed process of CLASS algorithm" in Supplementary Note 1 for detailed explanation on CASS and CLASS). Software-based AO via CLASS increased image intensity and SNR of CASS images. This effect of AO correction on the reflection images was much less dramatic than SMLM images (Figs. 3a, 3f, 4j, 4m). This is mainly because SMLM is much more prone to aberration than reflectance imaging due to its reliance on extremely weak single-molecule signals. In many cases, tissue aberrations were well resolved even when reflectance images hardly showed any recognizable structures. For example, some reflectance images looked like just speckle noises rather than specific biological structures as in **a**, **f**, **g**, and **h**. However, the same reflectance images were obtained for the repeated imaging of the same area. It suggests that these noise-like images originated from real sample structures. In some other cases, reflectance images revealed recognizable structures. For example, some of the dark, round patches observed in **b** and **e** might be blood vessels traversing in an out-of-plane direction. Blood vessels are quite frequently observed in LED transmission imaging. Sometimes, bright branch-like lines were observed (**b**-**e**). They are lipid-rich, highly reflective myelinated axons.

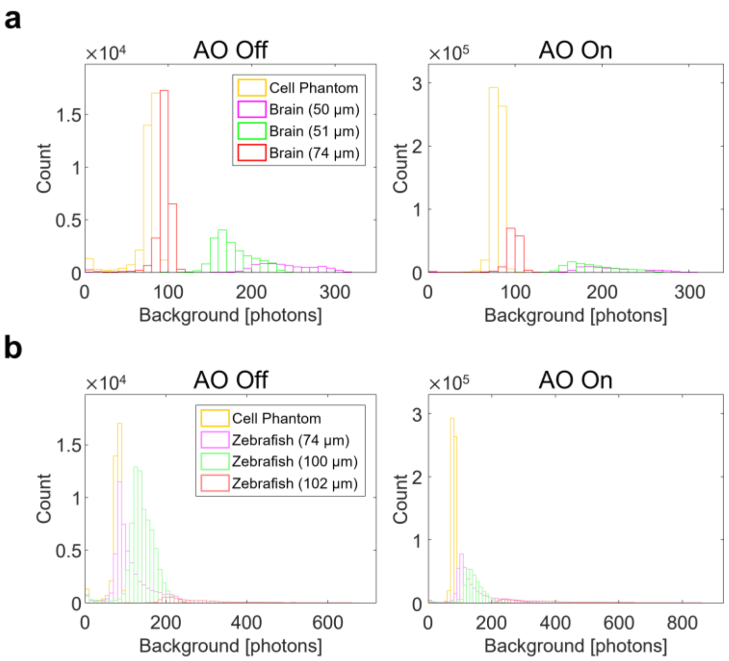

Supplementary Figure 6. **Histograms of background fluorescence for various samples.** **a**, **b** Background fluorescence per emission PSF obtained by ThunderSTORM analysis for a cell with an aberration layer, brain tissues (**a**), and whole zebrafish larvae (**b**). Cell phantom data was shown in both **a** and **b** for reference. Overall, background fluorescence was higher for brain tissues and whole zebrafish than cells. When imaging whole zebrafish, the fluorescence background generally increases as the imaging depth increases (**b**). As a result, there is a tendency that localization precision and the localization number become lower at deeper depths.

Sample	Figure	Depth [µm]	A_{RMS}	$w_{\mathrm{AO\ off}}$ [nm]	$w_{\mathrm{AO\ on}}$ [nm]	$\frac{S_{\mathrm{AO\ on}}}{S_{\mathrm{AO\ off}}}$	$\sigma_{\mathrm{AO\ off}}$ [nm]	$\sigma_{\mathrm{AO\ on}}$ [nm]	$\frac{N^{\mathrm{loc}}_{\mathrm{AO\ on}}}{N^{\mathrm{loc}}_{\mathrm{AO\ off}}}$
**Abrr.-Free Cell**	S3	-	-	x: 551<br>y: 579	x: 452<br>y: 494	1.01	Whole Area		
							25	23	1.02
**Cell + Abrr. Layer**	2c, d	-	2.22	x': 1430<br>y': 1032	x: 380<br>y: 444	4.27	Whole Area		
							51	22	14.8
							Yellow Box		
							57	22	25.5
							Green Box		
							83	23	14.2
**150-µm-Thick Mouse Brain**	3a	50	1.37	x: 668<br>y: 668	x: 413<br>y: 479	2.75	Whole Area		
							66	38	9.32
									Fig. 3b
									9.80
									Fig. 3c
									6.75
**200-µm-Thick Mouse Brain**	3f	74	0.983	x: 744<br>y: 698	x: 400<br>y: 475	3.92	Whole Area		
							60	37	4.79
									Fig. 3g
									12.8
									Fig. 3h
									12.2
									Fig. 3i
									12.1
**Zebrafish Cilia**	4d	82	2.14	x': 1077<br>y': 1061	x: 537<br>y: 517	1.93	Whole Area		
							52	34	1.62
**Zebrafish Commissural Tracts**	4g	100	3.08	x: 783<br>y: 662	x: 418<br>y: 489	1.17	Whole Area		
							67	38	1.02
**Zebrafish Nascent Myelin Sheath**	4j	52	2.13	x: 619<br>y: 659	x: 419<br>y: 434	3.34	Whole Area		
							53	35	8.59
									Arrowhead in Fig. 4j
									37.4
**Zebrafish Mauthner Axon**	4m	102	2.64	x': 496<br>y': 2006	x: 403<br>y: 458	8.86	Whole Area		
							Unmea.	34	16.2

Supplementary Table 1. Summary of measured parameters for various samples. A_{RMS}: RMS amplitude of sample aberration after removing trivial Zernike modes (tilt and defocus modes). $w_{\mathrm{AO\ off}}$ & $w_{\mathrm{AO\ on}}$: FWHMs of ensemble-averaged single-molecule PSFs for AO-off and -on, respectively. $S_{\mathrm{AO\ off}}$ & $S_{\mathrm{AO\ on}}$: Strehl ratios of ensemble-averaged single-molecule PSFs with AO-off and -on, respectively**.** $\sigma_{\mathrm{AO\ off}}$ & $\sigma_{\mathrm{AO\ on}}$: AO-off and -on localization precisions, respectively. $N^{\mathrm{loc}}_{\mathrm{AO\ off}}$ & $N^{\mathrm{loc}}_{\mathrm{AO\ on}}$: AO-off and -on localization numbers, respectively. Note that the x'- and y'-direction were defined as the longest axis and its orthogonal axis of the ensemble-averaged PSF, respectively. Also, $\sigma_{\mathrm{AO\ off}}$ was unmeasurable in Fig. 4m due to insufficient localization number.

Supplementary Note 1

Details layout of experimental setup

This section describes the complete experimental setup, crucial alignment steps, and data acquisition procedures. The detailed layout of the experimental setup is shown in Supplementary Figure 7. In the CLASS setup, we used a superluminal laser diode as a light source. Due to its short coherence length (~40 μm), it enabled time-gated detection in the interferometry. This provided depth gating defining the depth of interest. Its wavelength was 678 nm, which lies near the center wavelength of fluorescence from Alexa Fluor 647 used for SMLM imaging. The 678 nm laser beam traveled through a 2-axis galvanometer mirror (GV). Note here that gray rectangles without name tags represent mirrors. The beam was then divided into two branches at BS1. One of them, called the sample beam (SB), was delivered to the sample through BS2, FM1, FM2, TL, and OL1 for widefield illumination. Its beam size at the sample was set as ~100 μm in radius. Then, SB retraced the same path back through OL1, TL, FM2, FM1, and BS2. Afterward, it traveled toward sCMOS camera through BS4. Meanwhile, the other beam, called the reference beam (RB), traveled toward the reference mirror through BS3. The reference mirror was attached to an automated translation stage for time-gated imaging by adjusting the reference beam path length. This was required because interference only occurs when the optical path length difference between SB and RB is within the coherence length. After being reflected from the reference mirror, RB retraced the same path back and traveled toward the grating through BS3. Passing through the grating, RB was diffracted into several branches. Among them, only the first-order branch was selected for oblique incidence of RB at the camera plane. The other branches were discarded by blocking with an iris. The first-order RB traveled toward the sCMOS camera through BS4. At the camera plane, SB and RB formed an interference pattern when the length difference between them was shorter than the coherence length. The first-order diffraction from the grating was used to set the temporal front of RB parallel to the camera plane. In case RB is tilted by rotating a mirror in the RB path, the tilt of the temporal front disrupts the formation of the interference pattern with SB[6].

The CLASS setup was designed for confocal reflectance imaging as well as interferometric reflectance imaging. Switching to confocal mode was accomplished by laying down the FM1, raising up BS5, and moving the sCMOS camera in front of OL2. The sCMOS camera was attached to a detachable magnetic base for freely changing its location. In confocal mode, RB splitted at BS1 traveled toward the sample through BS3, FM2, TL, and OL1 for point illumination. After being reflected from the sample, SB retraced the same path back and traveled toward the sCMOS camera through GV, BS5, and OL2. Confocal reflectance imaging allows us to navigate the specimen and find the structure of interest in real-time.

In the SMLM setup, the activation and two excitation beams of wavelength 405 nm, 488 nm, and 647 nm, respectively, were coupled by BS6 and DM1. They traveled toward the sample through DM2, DM3, FM2, TL, and OL1 for widefield illumination. The emission beam from the sample traveled through OL1, TL, FM2, and DM3. It was then split into two branches at PBS1. One of them with a horizontal polarization was reflected from the SLM and phase-modulated for aberration correction. The other traveled toward the EMCCD through air without passing through the SLM. Both beams were combined by PBS2, traveling toward the EMCCD camera. They simultaneously arrived at spatially separated regions on the camera plane, enabling real-time comparison of AO-off and -on image frames.

The LED beam of a wavelength of 505 nm was used for low-magnification transmission imaging. It assisted in determining where to image. The back-illuminated LED beam passed through the sample and traveled toward a CCD camera through OL1, TL, FM2, DM3, and DM2.

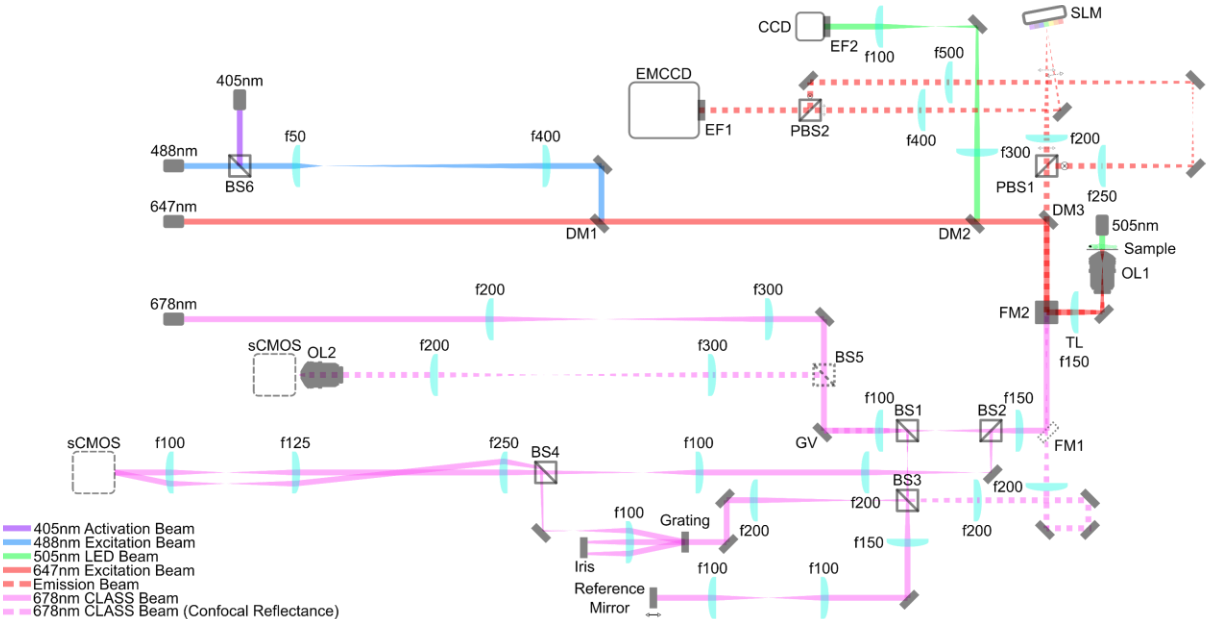

Supplementary Figure 7. Detailed layout of optical setup. PBS1-2: polarizing beam splitters, BS1-6: beam splitters, FM1-2: flip mirrors, OL1-2: objective lenses, TL: tube lens, GV: 2-axis galvanometer mirror, DM1-3: dichroic mirrors, EF1-2: emission filters, gray rectangles without name tags: mirrors, and fL_f (L_f = 100, 125, …): lenses of focal L_f in mm. One port of the commercial inverted microscope was devoted to CLASS imaging, and the other to SMLM imaging. Both setups share the sample, the objective (OL1), and the tube lens (TL). Also, they were switchable from each other using a built-in flip mirror (FM2) of the microscope. All lens pairs were used for either relaying images or adjusting beam size.

Preparation of aberration correction

As described above, the emission beam is split into two branches. For the fair comparison of AO-off and -on images, both images should have similar localization precision and photon number when there are no aberrations. To verify this, five different 200-nm-diameter fluorescent beads were imaged over 2,000 frames. Note that no aberrating layer was used in this test. For the test, the SLM was used as a mirror by setting all SLM pixels to the same phase values. Because SLM-unmodulated ("No SLM" in Supplementary Figure 8) and -modulated ("Through SLM" in Supplementary Figure 8) images were simultaneously recorded, ThunderSTORM analysis yielded 10 SMLM images for five beads. Half of them were constructed from SLM-unmodulated images, and the others from SLM-modulated images. Both SLM-unmodulated and SLM-modulated images yielded almost the same lateral localizations (σ_x and σ_y) and photon numbers. This justifies a fair comparison of AO-off and -on images.

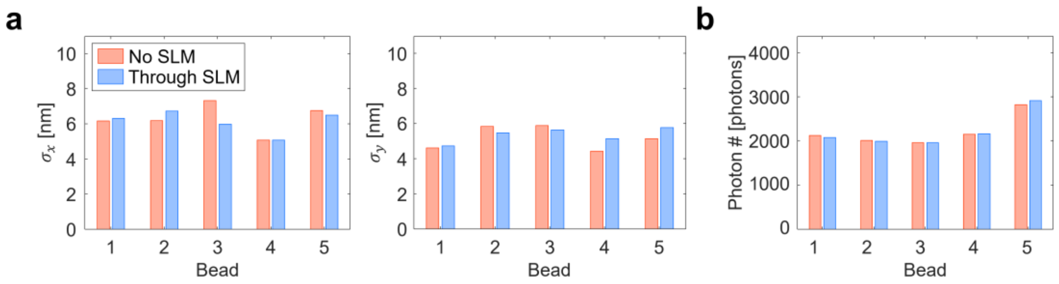

Supplementary Figure 8. Comparison of SLM-unmodulated and -modulated images. Both SLM-unmodulated and -modulated images of 200-nm-diameter fluorescent beads without aberration layer were compared while no pattern was displayed on the SLM. **a**, **b** Bar graphs of lateral localization precisions (**a**) and photon number (**b**) for SMLM images of 5 different beads. 2000 frames were obtained for each bead. No SLM: SLM-unmodulated image, Through SLM: SLM-modulated image.

Next, we introduce a procedure to spatially match the aberration correction map displayed on the SLM with the objective pupil of the emission beam (Supplementary Figure 9). The first step was to match their center positions. This process was accomplished by illuminating a mirror with a normally incident plane wave. Then, the excitation beam reflected from the mirror was focused on the center of the objective pupil. At the same time, the beam was focused on a certain position on the SLM display. That is to say, the focused beam position on the SLM is conjugated with the center of the objective pupil. To find this focused beam position, a grating pattern was displayed on the SLM (Supplementary Figure 9a). On the grating pattern, we inserted a small square area with an equal phase value (Supplementary Figure 9b). It served as a mirror patch. The pitch of the grating pattern was set fine enough that light impinging on it was diffracted away from the collection angle of the relay lenses, thereby unable to reach the camera view field. Therefore, the beam entering the mirror patch could only arrive at the camera, contributing to camera readout. The mirror patch was raster-scanned while recording the camera intensity (Supplementary Figure 9c). The intensity was maximum when the mirror patch coincided with the focused beam indicated by a red circle in Supplementary Figure 9b. That is, the center position of the aberration correction map was determined as the position of the mirror patch at which the intensity is maximum. In these measurements, the size of the mirror patch was set to ~100 µm. Too small or large mirror patch results in either negligible intensity variation or poor positioning precision.

The second step was to determine the diameter of the aberration correction map displayed on the SLM plane. To this end, confluent fluorescent beads were loaded on the sample plane. Then, the emission beam formed a solid circle on the SLM plane indicated by a red circle in Supplementary Figure 9d. The diameter of the aberration correction map should be equal to that of this emission beam for precise correction. To measure its diameter, the scanning method explained above was used again, but a mirror slab was scanned instead of a mirror patch. In the beginning, a grating pattern was displayed on the SLM. Then, starting from the left side, the grating pattern was replaced by a mirror slab (Supplementary Figure 9d). The mirror slab consisted of pixels with the same phase values. While expanding the mirror slab from left to right, the intensity of the camera image was recorded (Supplementary Figure 9e). At some point, the emission beam started to overlap with the mirror slab. Then, the intensity began to increase. The intensity was saturated when the mirror slab fully covered the emission beam. The emission beam diameter could be evaluated as the length of the region where the intensity was increased.

Using the calibrated results explained above, the display region of the aberration correction map was determined (gray circle in Supplementary Figure 9f).

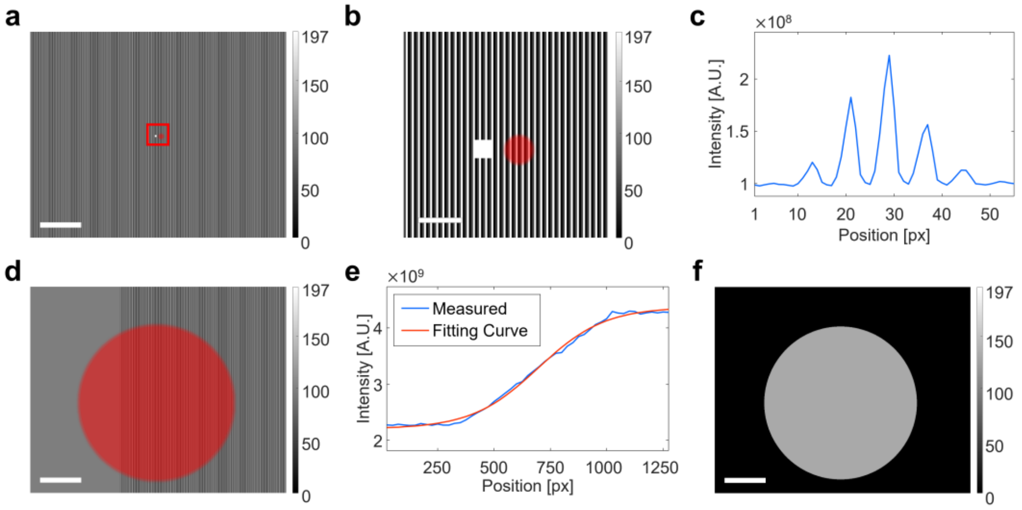

Supplementary Figure 9. Aberration correction map positioning procedure. a, **b** Grating pattern with a small mirror patch displayed on the SLM (**a**) and a magnified view of red box (**b**). The white box is a small mirror patch. The solid red circle depicts the focused beam on the SLM plane. Color bars indicate SLM pixel values. Scale bars indicate 2.5 mm (**a**) and 250 μm (**b**). **c** Intensity of recorded image during mirror patch scanning. **d** Grating pattern with a mirror slab. The solid red circle is the emission beam on the SLM plane. Color bar indicates SLM pixel values. Scale bar indicates 2.5 mm. **e** Intensity of recorded image during mirror slab scanning. A.U. indicates arbitrary unit. **f** Display region of the aberration correction map. A gray circle shows where correction maps to be displayed. Color bar indicates SLM pixel values. Scale bar indicates 2.5 mm.

The orientation and tilt of the aberration correction map displayed on the SLM were determined by a simple test (Supplementary Figure 10). The correction map can be rotated counterclockwise by 0°, 90°, 180°, and 270° when displayed on the SLM. Also, it can be flipped horizontally. Hence, there are 8 cases in total. Note that vertical flip results in the duplicated cases because it is identical to 180° counterclockwise rotation and horizontal flip. Then, the correct combination can be found by trying to correct the aberration of fluorescent beads with an aberrating layer while displaying eight kinds of aberration correction maps. In our system, the aberration correction worked when the aberration correction map was counterclockwise rotated by 270° with no flip.

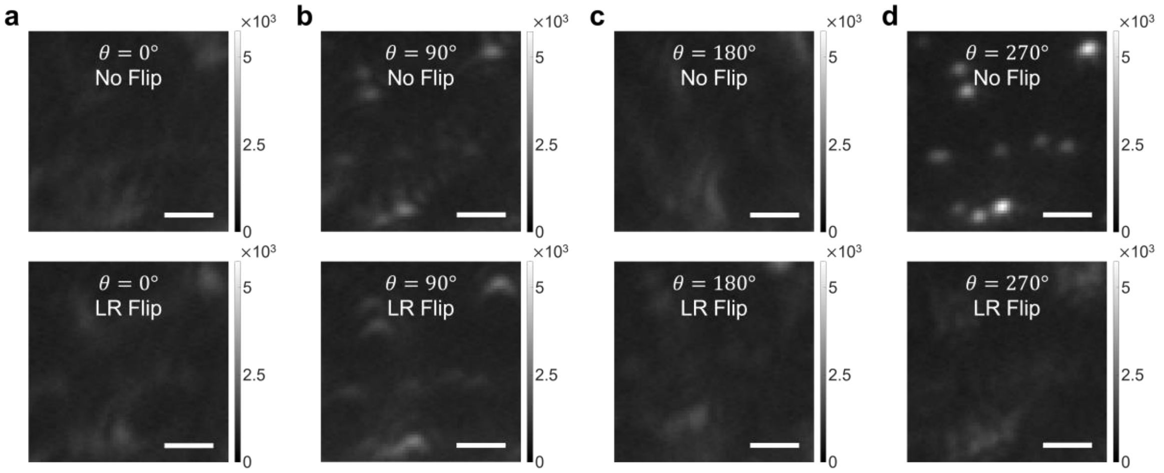

Supplementary Figure 10. Determination of orientation and tilt of displaying aberration correction map. a-d Diffraction-limited fluorescence images of 200-nm-diameter beads for eight different orientation and tilt angles of the aberration correction map displayed on the SLM. Each was corrected by displaying the aberration correction map rotated by θ counterclockwise on the SLM (θ = 0°, 90°, 180°, and 270°). For each degree, the aberration correction map was displayed without (top row) or with a left-to-right flip (bottom row). Scale bars indicate 2 μm.

The optics layout of SMLM is different from that of the CLASS microscope except for the sample, the objective lens, and the tube lens (Supplementary Figure 7). Therefore, it was necessary to separately correct for SMLM system aberration before correcting the sample-induced aberration identified by the CLASS (Supplementary Figure 11). The system aberration A of SMLM can be written as a linear combination of Zernike modes ($A = \sum_n c_n Z_n$). Here, c_n is the coefficient of the nth Zernike mode Z_n. If each Zernike mode is displayed on the SLM while changing its coefficient, the aberration experienced by the light through the SLM changes according to the coefficient. Then, the PSF intensity is generally maximum when the aberration is minimum. Based on this property, the coefficient c_n of each mode Z_n consisting of the system aberration A can be determined.

To this end, we tuned the coefficient c_n of each mode Z_n in such a way as to maximize the intensity of the PSF. Note that the first and second Zernike modes were excluded because they only spatially shift PSF rather than change its shape and intensity. Also, the PSF was indirectly measured by imaging a 200-nm diameter fluorescent bead. Starting from the Zernike mode 3, the measured PSF intensity was fitted with a double Gaussian function to find c_3 maximizing the PSF intensity (Supplementary Figure 11a). While displaying $c_3 Z_3$ on the SLM, we added Zernike mode 4 and tuned its coefficient to find c_4 maximizing the PSF intensity (Supplementary Figure 11b). The same process was repeated up to Zernike mode 20 to find SMLM system aberration (Supplementary Figure 11c). Correcting system aberration increased PSF intensity and reduced PSF widths in both x- and y-direction (Supplementary Figure 11d-e). With AO, PSF FWHM (Supplementary Figure 11e) was slightly larger than the ideal FWHM $w_{\text{no abrr.}}$, which is calculated as

$$w_{\text{no abrr.}} \simeq 0.61 \frac{\lambda}{\text{NA}} = 0.61 \frac{670 \text{ nm}}{1.2} \simeq 341 \text{ nm}.$$

This suggests that weak SMLM system aberration remained even with AO. However, it was no trouble at all considering that sub-diffraction structures were well resolved with AO in our study.

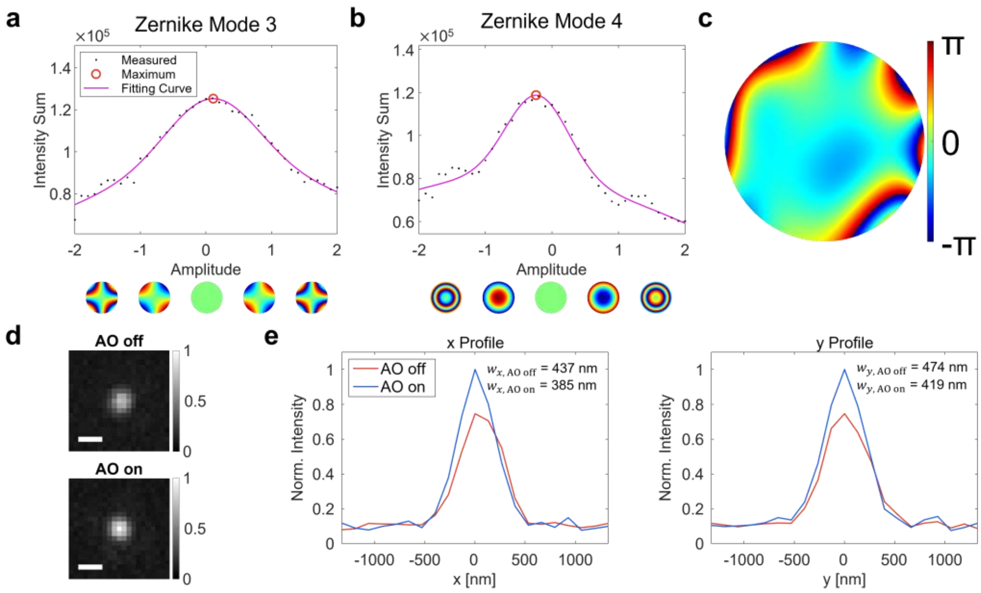

Supplementary Figure 11. SMLM system aberration correction. a, **b** Intensity vs. mode coefficient plots for Zernike modes 3 (**a**) and 4 (**b**), respectively. **c** Identified SMLM system aberration. **d** Diffraction-limited fluorescence images of a 200-nm-diameter fluorescent bead without and with AO, respectively. Images are normalized with AO-on image. Color bars indicate normalized image intensity. Scale bars indicate 500 nm. **e** Line profiles of AO-off and -on images in **d**.

Experimental procedure

The imaging process of our AO-SMLM is described in Supplementary Figure 12. For CLASS imaging, two sets of interference images were recorded (Supplementary Figure 12a). They were composed of angle-scanned interference image sets of a mirror and the sample at the sample plane, respectively. Then, a reflection matrix R was constructed using these two sets of images (Supplementary Figure 12b). By applying the CLASS algorithm to R, we obtained a sample-induced aberration map (Supplementary Figure 12c). The next step was checking the validity of the identified aberration map. It was accomplished by confirming that the software-based aberration correction well recovered the reflection image reconstructed from R (Supplementary Figure 12d). Then, the opposite phase of the calculated aberration was displayed on the SLM to correct the aberration experienced by the single-molecule emission PSFs.

After that, we acquired raw images of blinking single molecules (Supplementary Figure 12e). As described above, the emission beam was split into two branches. One traveled through air without passing through the SLM and was recorded as AO-off raw images. The other traveled through the SLM and was recorded as AO-on raw images. Both AO-off and -on beams arrived at two different regions of the camera sensor for simultaneous recording. Lastly, both of these raw images were processed by ThunderSTORM software for constructing SMLM images and further post-processing (Supplementary Figure 12f).

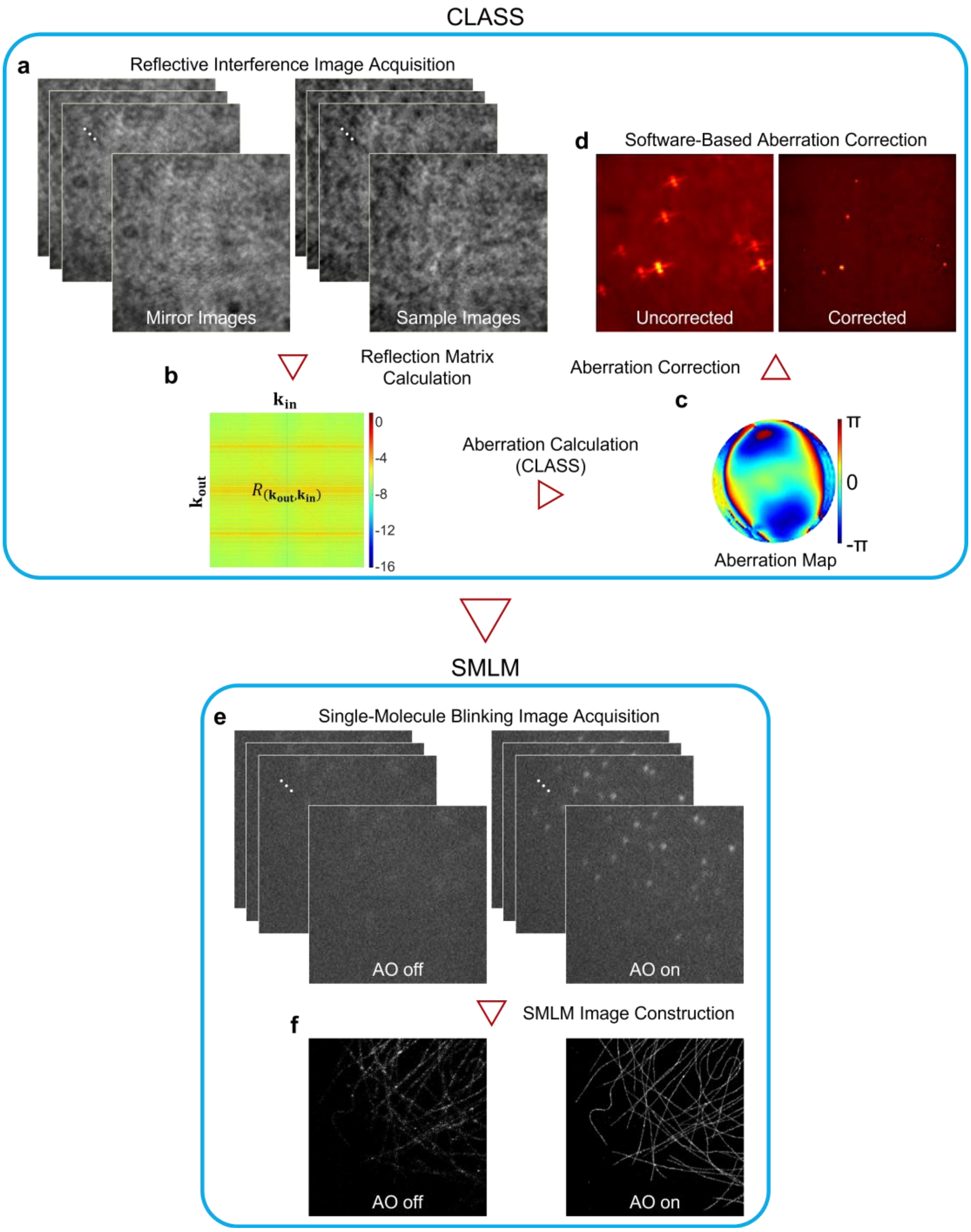

Supplementary Figure 12. Imaging process. a-d CLASS microscopy process. **e**, **f** SMLM imaging process.

Detailed process of CLASS algorithm

A rigorous description of CLASS starts from mathematically expressing interference. To this end, let $E_{\mathrm{S}}(\mathbf{r};\mathbf{k}_{\mathrm{in}})$ and $E_{\mathrm{R}}(\mathbf{r};\mathbf{k}_{\mathrm{in}})e^{-i\mathbf{k}_{\mathrm{G}}\cdot\mathbf{r}}$ be the electric fields of the sample beam (SB) and reference beam (RB), respectively, at position $\mathbf{r}=(x,y)$ on the camera plane. Here, E_{S} and E_{R} are complex numbers, and $\mathbf{k}_{\mathrm{in}}$ is the transverse wavevector set by illumination angle for each galvanometer mirror scanning position. The phase term $\mathbf{k}_{\mathrm{G}}\cdot\mathbf{r}$ is introduced because the RB is chosen from the first-order diffraction by a diffraction grating with spatial frequency $\mathbf{k}_{\mathrm{G}}$. Since RB is simultaneously rotated in sync with SB, the reference wave can be written as $E_{\mathrm{R}}(\mathbf{r};\mathbf{k}_{\mathrm{in}})=e^{i\mathbf{k}_{\mathrm{in}}\cdot\mathbf{r}}$. On the camera plane, interference occurs if the optical path length difference between SB and RB is smaller than the coherence length of the light source. Since a camera detects light intensity rather than the field itself, a camera image composed of SB and RB (Supplementary Figure 13a) is written by the absolute square of their field sum. That is,

$$\begin{aligned} I(\mathbf{r};\mathbf{k}_{\mathrm{in}}) &= \left|E_{\mathrm{S}}(\mathbf{r};\mathbf{k}_{\mathrm{in}})+E_{\mathrm{R}}(\mathbf{r};\mathbf{k}_{\mathrm{in}})e^{-i\mathbf{k}_{\mathrm{G}}\cdot\mathbf{r}}\right|^2 \\ &= |E_{\mathrm{S}}(\mathbf{r};\mathbf{k}_{\mathrm{in}})|^2+|E_{\mathrm{R}}(\mathbf{r};\mathbf{k}_{\mathrm{in}})|^2+E_{\mathrm{S}}(\mathbf{r};\mathbf{k}_{\mathrm{in}})E_{\mathrm{R}}^*(\mathbf{r};\mathbf{k}_{\mathrm{in}})e^{i\mathbf{k}_{\mathrm{G}}\cdot\mathbf{r}}+E_{\mathrm{S}}^*(\mathbf{r};\mathbf{k}_{\mathrm{in}})E_R(\mathbf{r};\mathbf{k}_{\mathrm{in}})e^{-i\mathbf{k}_{\mathrm{G}}\cdot\mathbf{r}}. \end{aligned} \quad (1)$$

To extract the interference term $E_{\mathrm{S}}(\mathbf{r};\mathbf{k}_{\mathrm{in}})E_{\mathrm{R}}^*(\mathbf{r};\mathbf{k}_{\mathrm{in}})$, we take 2-D Fourier transform of the interference image (Supplementary Figure 13b). We then select and crop the spectral component $\mathcal{F}\{E_{\mathrm{S}}(\mathbf{r};\mathbf{k}_{\mathrm{in}})E_{\mathrm{R}}^*(\mathbf{r};\mathbf{k}_{\mathrm{in}})\mathrm{e}^{i\mathbf{k}_{\mathrm{G}}\cdot\mathbf{r}}\}$ indicated by a red circle whose center is set by $\mathbf{k}_{\mathrm{G}}$. Cropped component is sent to a new coordinate system where its origin is $\mathbf{k}_{\mathrm{G}}$ (Supplementary Figure 13c). This means that $\mathbf{k}_{\mathrm{G}}$ vanishes in the new coordinates. In this new coordinate system, the selected spectral component is written as $\mathcal{F}\{E_{\mathrm{S}}(\mathbf{r};\mathbf{k}_{\mathrm{in}})E_{\mathrm{R}}^*(\mathbf{r};\mathbf{k}_{\mathrm{in}})\}$. Taking the inverse Fourier transform, we obtain the $E_{\mathrm{S}}(\mathbf{r};\mathbf{k}_{\mathrm{in}})E_{\mathrm{R}}^*(\mathbf{r};\mathbf{k}_{\mathrm{in}})$ (Supplementary Figure 13d).

The next step is to obtain $E_{\mathrm{S}}(\mathbf{r};\mathbf{k}_{\mathrm{in}})$ from the measured field $E_{\mathrm{S}}(\mathbf{r};\mathbf{k}_{\mathrm{in}})E_{\mathrm{R}}^*(\mathbf{r};\mathbf{k}_{\mathrm{in}})=E_{\mathrm{S}}(\mathbf{r};\mathbf{k}_{\mathrm{in}})|E_R^*|e^{-i\mathbf{k}_{\mathrm{in}}\cdot\mathbf{r}}$. Removing the factor $e^{-i\mathbf{k}_{\mathrm{in}}\cdot\mathbf{r}}$ requires precise knowledge of $\mathbf{k}_{\mathrm{in}}$. This process is accomplished by imaging a scattering medium with the same incidence angles $\mathbf{k}_{\mathrm{in}}$. The scattered light by the scattering medium fully covers the pupil in the Fourier space (Supplementary Figure 13e). Since this pupil rotates as the incidence angle $\mathbf{k}_{\mathrm{in}}$ changes, $\mathbf{k}_{\mathrm{in}}$ can be obtained from the center of the pupil. Then, the factor $e^{-i\mathbf{k}_{\mathrm{in}}\cdot\mathbf{r}}$ is removed from each measured spectrum $E_{\mathrm{S}}(\mathbf{r};\mathbf{k}_{\mathrm{in}})E_{\mathrm{R}}^*(\mathbf{r};\mathbf{k}_{\mathrm{in}})$ in Supplementary Figure 13c by multiplying $e^{i\mathbf{k}_{\mathrm{in}}\cdot\mathbf{r}}$.

We measure two sets of $E_{\mathrm{S}}(\mathbf{r};\mathbf{k}_{\mathrm{in}})$ for various $\mathbf{k}_{\mathrm{in}}$, one with a mirror on the sample plane and the other by placing the sample of interest, respectively. After that, two matrices, P and T, are constructed to calculate the aberration (Supplementary Figure 13f). The Fourier-transformed mirror image for the $\mathrm{n^{th}}$ incidence angle is vectorized and becomes the $\mathrm{n^{th}}$ column of a matrix P. Similarly, the vectorized Fourier-transformed sample image for the $\mathrm{n^{th}}$ incidence angle composes the $\mathrm{n^{th}}$ column of matrix T. Then the reflection matrix $R_{(\mathbf{k}_{\mathrm{out}},\mathbf{k}_{\mathrm{in}})}$ is obtained from the relation,

$$R_{(\mathbf{k}_{\mathrm{out}},\mathbf{k}_{\mathrm{in}})} \equiv T\times P^{-1}, \quad (2)$$

where P^{-1} denotes the pseudoinverse matrix of P.

The expression $R_{(\mathbf{k}_{\mathrm{out}},\mathbf{k}_{\mathrm{in}})}$ means that the row of R correspond to incidence angle $\mathbf{k}_{\mathrm{in}}$ and their columns correspond to output angle $\mathbf{k}_{\mathrm{out}}$ (Supplementary Figure 13g). Simply speaking, the jth column of $R_{(\mathbf{k}_{\mathrm{out}},\mathbf{k}_{\mathrm{in}})}$ is a vectorized Fourier-transformed sample image for the jth illumination angle $\mathbf{k}_{\mathrm{in}}^{(j)}$. $R_{(\mathbf{k}_{\mathrm{out}},\mathbf{k}_{\mathrm{in}})}$ shows every possible Fourier-transformed sample image for each illumination angle $\mathbf{k}_{\mathrm{in}}^{(j)}$. Here, $j=1,2,\ldots,N$ with N the number of incidence angles. In other words, each column of the matrix contains the spatial frequency spectrum of the sample wave. It can be described by the following relation:

$$\tilde{E}(\mathbf{k}_{\mathrm{out}};\mathbf{k}_{\mathrm{in}})=\sqrt{\gamma}P_{\mathrm{out}}^{\mathrm{a}}(\mathbf{k}_{\mathrm{out}})\tilde{O}(\mathbf{k}_{\mathrm{out}}-\mathbf{k}_{\mathrm{in}})P_{\mathrm{in}}^{\mathrm{a}}(\mathbf{k}_{\mathrm{in}})+\sqrt{\beta}\tilde{E}_M(\mathbf{k}_{\mathrm{out}};\mathbf{k}_{\mathrm{in}}). \quad (3)$$

Here, β and γ are intensities of multiple- and single-scattered waves, respectively[7]. $P_{\mathrm{in}}^{\mathrm{a}}(\mathbf{k}_{\mathrm{in}})=P(\mathbf{k}_{\mathrm{in}})\exp[i\phi_{\mathrm{in}}(\mathbf{k}_{\mathrm{in}})]$ and $P_{\mathrm{out}}^{\mathrm{a}}(\mathbf{k}_{\mathrm{out}})=P(\mathbf{k}_{\mathrm{out}})\exp[i\phi_{\mathrm{out}}(\mathbf{k}_{\mathrm{out}})]$ are respectively the input and output pupil functions in the presence of aberrations. $\phi_{\mathrm{in}}(\mathbf{k}_{\mathrm{in}})$ and $\phi_{\mathrm{out}}(\mathbf{k}_{\mathrm{out}})$ are the input and output aberrations, respectively, and $P(\mathbf{k})$ describes the ideal pupil function, which is unity when $|\mathbf{k}|\le k_0\alpha$ and zero otherwise. Here, α is the numerical aperture of the objective lens. Our CLASS algorithm is unique in that it finds $\phi_{\mathrm{in}}(\mathbf{k}_{\mathrm{in}})$, $\phi_{\mathrm{out}}(\mathbf{k}_{\mathrm{out}})$, and $P(\mathbf{k})$ out of the reflection measurements.

The detailed process of CLASS to process $R_{(\mathbf{k}_{\mathrm{out}},\mathbf{k}_{\mathrm{in}})}$ for calculating sample-induced aberration is as follows. For this, each column of R is shifted by $\mathbf{k}_{\mathrm{in}}$ and add them all together. This coherent sum increases both the single-

scattering signal preserving the sample information and the multiple-scattering noise. However, as images are coherently added, the single-scattering signal increases faster than the multiple-scattering one by a factor of the number N of incidence angles. Inverse Fourier transform of this coherent sum gives an image whose single-scattering signal is significantly intensified. This is the process termed collective accumulation of single-scattered waves (CASS)[8]. However, the resultant CASS image still looks blurred (Supplementary Figure 13h). This is due to the angle-dependent phase retardation known as an aberration. It undermines the proper accumulation of the single-scattering signal. In this example, the sample was intentionally defocused to introduce the well-known defocus aberration.

To correct the aberration, each column of $R_{(\mathbf{k}_{out},\mathbf{k}_{in})}$ is multiplied by a phase value ranging from 0 to 2π. Then, the total intensity of the CASS image is maximized by adjusting phase values multiplied. Repeating this process for all the columns gradually increases the total image intensity. A similar process is made to the rows of $R_{(\mathbf{k}_{out},\mathbf{k}_{in})}$ to correct aberrations in the reflection beam path. After a few iterations, the intensity enhancement is saturated (Supplementary Figure 13i). This process is termed the closed loop accumulation of single scattering (CLASS) algorithm[9]. At this moment, aberration map is obtained by reshaping phase array applied to the rows of $R_{(\mathbf{k}_{out},\mathbf{k}_{in})}$ into a square form (Supplementary Figure 13j). Finally, correcting this aberration yields an aberration-corrected CASS image (Supplementary Figure 13k).

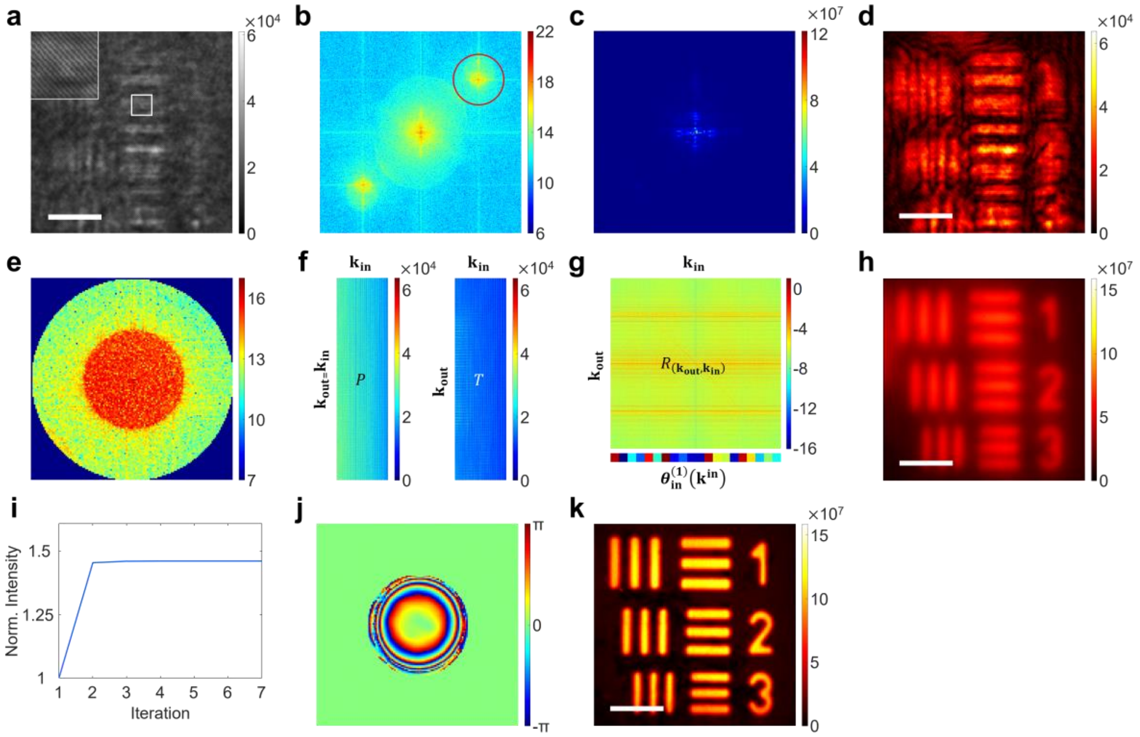

Supplementary Figure 13. Detailed process of CLASS microscopy. a A raw interference image of a USAF resolution target. Inset shows magnified view of the boxed region. It clearly exhibits diagonal interference fringe. Color bar indicates camera readout. Scale bar indicates 5 μm. **b**, **c** Fourier transform (**b**) of the interference image in **a** and selected spectral component (**c**) indicated by a red circle in **b**. Color bar indicates phase value. **d** Amplitude of the inverse Fourier transform of selected spectral component in **c**. There is also the phase map, but not shown here for brevity. Color bar indicates image intensity. Scale bar indicates 5 μm. **e** Pupil fully covered by multiple scattering induced by a scattering medium. Color bar indicates phase value. **f**, **g** P and T matrices (**f**) whose nth column is the vectorized Fourier-transformed image of a mirror and the sample, respectively. Reflection matrix R (**g**). Color bars indicates phase values. **h** CASS image. Color bar indicates image intensity. Scale bar indicates 5 μm. **i** Intensity enhancement during CLASS iteration. **j** Aberration map identified by CLASS. Color bar indicates phase value. **k** Aberration-corrected CASS image. Color bar indicates image intensity. Scale bar indicates 5 μm.

Supplementary Note 2

Comparison of CLASS-SMLM with the current state-of-the-art AO-SMLM

In this section, we present experimental and analytical results comparing the aberration correction capabilities of our CLASS-SMLM method with those of existing AO-SMLM methods. Our results showed that the previous AO-SMLM could correct aberrations up to around 1 rad in RMS wavefront distortion, as reported in the literature. However, it was not able to correct the same aberration map that our CLASS-SMLM could correct when the wavefront distortion exceeds well beyond 1 rad. These additional experiments confirmed the superior performance of our newly proposed approach.

We chose REALM[10], the latest modality in the field, as a point of reference. Following this earlier work, we prepared a monolayer of COS-7 cells and displayed a known aberration ϕ_{applied} on the spatial light modulator (SLM) in the emission beam path. We then used the REALM algorithm to find an aberration correction map. Essentially, the algorithm optimizes a designed image metric evaluated from blinking images of single molecules in the microtubules of COS-7 cells.

To check the fidelity of REALM, we measured the aberration correction map ϕ_{system} before applying ϕ_{applied} and ϕ_{total} after displaying ϕ_{applied}, respectively. Here, ϕ_{system} measures the aberration from the cells and SMLM setup. We compared the measured net aberration $\phi_{\mathrm{net}} = \phi_{\mathrm{total}} - \phi_{\mathrm{system}}$ to ϕ_{applied}. If the measurement were perfect, ϕ_{net} would be equal to ϕ_{applied}.

To assess the degree of aberrations that REALM can handle, we gradually increased the RMS wavefront distortion of ϕ_{applied} from 1 rad to ~3 rad. We verified that REALM could find the aberration map faithfully when RMS wavefront distortion was 1 rad (Supplementary Figure 14a). In this case, the aberration map was made of low-order Zernike modes (up to the 15th mode). ϕ_{applied} and ϕ_{net} look almost the same with a correlation of 0.970.

Under the same experimental condition of the cell monolayer sample, we applied the aberrations from brain tissues and zebrafish. We displayed an aberration map from the mouse brain tissue (0.983 rad in RMS wavefront distortion) and obtained a correlation of 0.895 between ϕ_{applied} and ϕ_{net} (Supplementary Figure 14b). There was a slight decrease in correlation because real sample aberrations include high-order Zernike modes, which are not dealt with in the REALM algorithm. REALM showed worse performance for aberration maps with increased wavefront distortion. For zebrafish aberrations with RMS wavefront distortions of 2.14 rad and 3.08 rad, the correlations were 0.654 (Supplementary Figure 14c) and 0.133 (Supplementary Figure 14d), respectively.

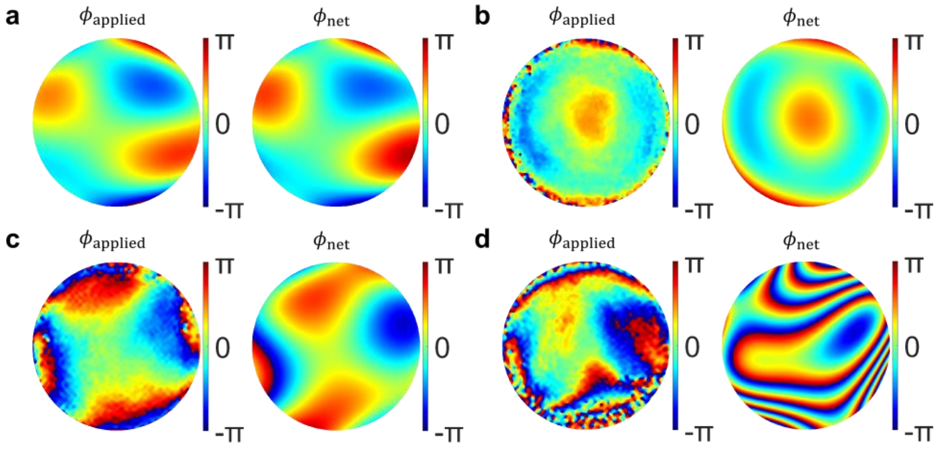

Supplementary Figure 14. Performance evaluation of REALM in finding the applied aberrations. Aberration maps ϕ_{applied} applied on the SLM and ϕ_{net} found by the REALM for four different levels of RMS wavefront distortions. **a** ϕ_{applied} is an artificial aberration made of Zernike modes used in REALM. Its RMS wavefront distortion is 1 rad. **b-d** Applied aberrations ϕ_{applied} are those in Fig. 3f (**b**; 0.983 rad), Fig. 4d (**c**; 2.14 rad), and Fig. 4g (**d**; 3.08 rad). Correlations are C_{a} = 0.970, C_{b} = 0.895, C_{c} = 0.654, and $C_{\mathrm{d}} = 0.133$, where C_i represents the correlation between ϕ_{applied} and ϕ_{net} for i = **a**, **b**, **c**, or **d**.

As a next step, we conducted SMLM imaging of microtubules of COS-7 cells by correcting the applied aberration ϕ_{applied} with ϕ_{net} found by REALM (Supplementary Figure 15). In other words, we displayed residual aberration $\phi_{\text{residual}} = \phi_{\text{applied}} - \phi_{\text{net}}$ on the SLM. We observed a gradual reduction in localization number and resolving power with the increase of the RMS wavefront distortion of ϕ_{applied}. In particular, almost all the microtubules were missing for 3.08-rad zebrafish aberration (Supplementary Figure 15d). We would like to emphasize that our CLASS-SMLM worked well for this level of aberration even at a depth of 102 μm inside a whole zebrafish. On the contrary, REALM didn't work at all for the same aberration map for the cell monolayer samples where there were almost no depth-related signal loss and background fluorescence noise.

Note that we implemented REALM in a better setting than the original paper. Our aberration correction map was composed of 283,657 SLM pixels while the REALM paper used a deformable mirror (DM) with only 52 actuators[10]. As we analyzed in detail, the number of correction elements plays an important role in the aberration correction (see Supplementary Figure 17 for detailed discussion).

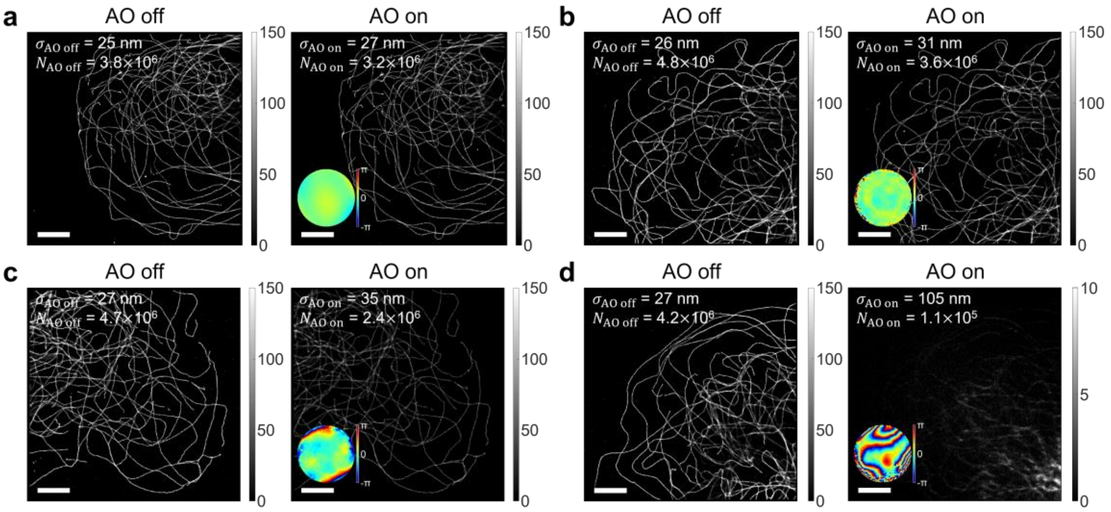

Supplementary Figure 15. SMLM imaging by the REALM for COS-7 cells with the applied aberration $\boldsymbol{\phi_{\text{applied}}}$. a-d AO-off and -on SMLM images. AO-off SMLM images were obtained without AO. AO-on SMLM images were obtained by displaying residual uncorrected aberration $\phi_{\text{residual}} = \phi_{\text{applied}} - \phi_{\text{net}}$ on the SLM. (ϕ_{applied} and ϕ_{net} shown in Supplementary Figure 14). RMS wavefront distortions of ϕ_{residual} were 0.246 rad (**a**), 0.570 rad (**b**), 1.22 rad (**c**), and 5.98 rad (**d**). Insets indicate ϕ_{residual}. $\sigma_{\text{AO off}}$ and $\sigma_{\text{AO on}}$ represent AO-off and -on localization precisions. $N_{\text{AO off}}$ and $N_{\text{AO on}}$ represent AO-off and -on localization numbers. Color bars indicate localization numbers. Scale bars indicate 5 μm.

Lastly, we verified the performance of REALM by imaging whole zebrafish embryos. We found that REALM worked to the extent that the measured aberration was as mild as 1 rad in RMS wavefront distortion (Supplementary Figure 16a-c). While the aberration map identified by the REALM was not identical to the aberration map measured by CLASS, they have a reasonable correlation of 0.591. Referring to Supplementary Figure 14c and Supplementary Figure 15c (correlation was 0.654), this is high enough to explain the successful aberration correction by the REALM. However, when the aberration exceeded 2 rad in RMS wavefront distortion, the REALM didn't work at all (Supplementary Figure 16d-f). There were significant degradation in localization precision and a reduction in localization number. The aberration correction map by the REALM was completely different from that by the CLASS (Supplementary Figure 16e, f). Their correlation was only 0.0688. Moreover, multiple trials of the REALM on the same region resulted in finding different aberrations (their correlations < 0.3), which confirms that the REALM completely failed.

As was presented in the manuscript, our CLASS-SMLM worked well in imaging a whole zebrafish even when the aberration exceeds 3 rad in RMS wavefront distortion. Therefore, our CLASS-SMLM is superior to the previous AO-SMLM when it comes to complex tissue aberrations. Note here that it is intrinsically not possible to conduct SMLM imaging for the same spot by both REALM and CLASS-SMLM. Most of the dyes were photobleached during raw image acquisition with a REALM correction map, making it impossible to conduct

another round of image acquisition with a CLASS correction map. Therefore, we considered CLASS-identified aberration as a ground truth and compared it with the aberration map identified by REALM to see how far the REALM works properly.

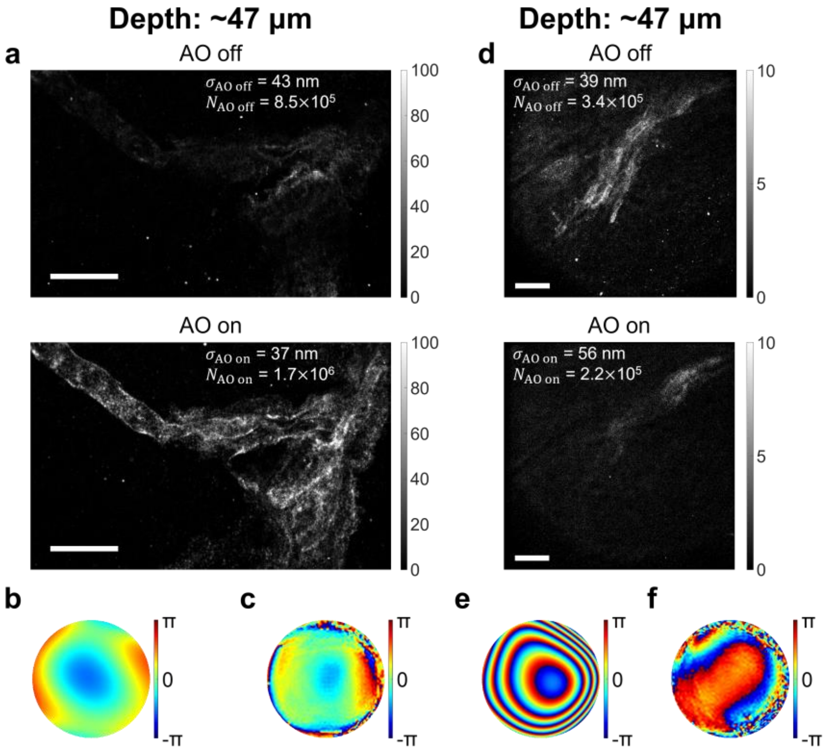

Supplementary Figure 16. SMLM images of oligodendrocytes in spinal cords of the 5-dpf whole zebrafish embryos by the REALM at a depth of 47 μm. a AO-off and -on SMLM images with REALM correction map in **b**. Scale bar indicate 5 μm. **b**, **c** Aberration correction maps found by REALM (**b**) and CLASS (**c**). **d**-**f** Same as **a**-**c**, respectively, but for a different sample. $\sigma_{\text{AO off}}$ and $\sigma_{\text{AO on}}$ represent AO-off and -on localization precisions. $N_{\text{AO off}}$ and $N_{\text{AO on}}$ represent AO-off and -on localization numbers. Color bars indicate localization numbers. Scale bar indicate 5 μm. The RMS wavefront distortions of the aberration correction maps are 0.850 rad (**b**), 1.66 rad (**c**), 7.05 rad (**e**), and 2.18 rad (**f**). The correlation between **c** and **d** is 0.591, and that between **e** and **f** is 0.0688.

Photoswitching to reduce PSF overlap

We used conventional SMLM employing photoswitching to minimize the overlapping of PSFs. For inducing photoswitching, an imaging buffer was added to the sample chamber (see "Imaging buffer preparation" section in Methods for detailed preparation of the imaging buffer). The light-induced reactions of Alexa Fluor 647 with thiol cause the repeated switching of dye molecules between off and on states[11,12]. In this process, some dye molecules are photobleached such that the spatial density of switched-on dyes continuously decreases. This leads to the reduction of the PSF overlap. Image acquisition gets started only after enough dye molecules are switched off and PSF overlap becomes negligible.

Aberration correction maps with different spatial frequency resolution

We investigated how SLM pixel number affects restored PSF. For PSF evaluation, we imaged 100-nm-diameter fluorescent beads covered with an aberrating layer. Aberration was measured using reflections from pre-attached 100-nm-diameter gold particles. Without AO, the PSF was highly distorted due to the presence of substantial aberration (Supplementary Figure 17a). Using CLASS-SMLM, we obtained the aberration correction map (bottom, Supplementary Figure 17b), whose RMS wavefront distortion was estimated to be 1.78 rad. By displaying this correction map on the SLM, we could obtain a clean and bright PSF (top, Supplementary Figure 17b). The number of SLM pixels used for this correction map was 283,657.

To verify the effect of the number of wavefront cells for aberration correction, we grouped several SLM pixels together and controlled them by designating their average value. We increased the number of pixels being grouped together on the x and y axis from 2^1 to 2^{10}. In other words, the spatial frequency resolution Δk of the aberration correction was varied from 2^1 to 2^{10} SLM pixels. Then, we recorded the aberration-corrected bead images while displaying individual correction maps (Supplementary Figure 17c-e). As expected, the width and intensity of the PSF were deteriorated with the increase of Δk (Supplementary Figure 17f-h). Notably, there was an abrupt increase of the PSF width from $\Delta k = 2^8$ SLM pixels (Supplementary Figure 17g) while the PSF intensity decreased steadily (Supplementary Figure 17h). This decrease was faster than normal bleaching (green curve in Supplementary Figure 17h), making it clear that this intensity decrease resulted from uncorrected residual aberration. As a point of reference, we indicated the achievable level of aberration correction by the deformable mirror as black vertical lines in Supplementary Figure 17g, h given its small number of actuators.

In conclusion, the reduction in the wavefront cells has a detrimental effect on the sharpness and brightness of the PSF. On the one hand, there is a specific number of wavefront cell number that can recover PSF width close to the diffraction limit for a given complexity of the aberration. On the other hand, the brightness of the PSF decreases steadily with the reduction of the wavefront cell number. Considering the importance of the PSF intensity in the localization of single molecules especially for thick tissues, it is better to use as many wavefront cells as possible for optimal aberration correction.

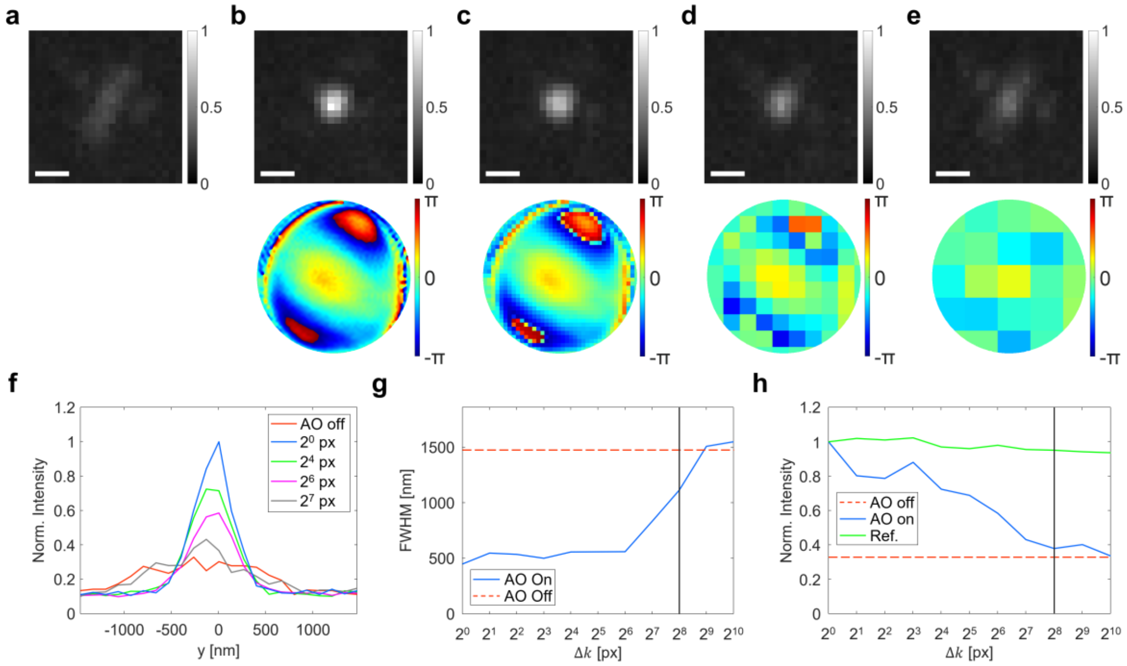

Supplementary Figure 17. Aberration correction with correction maps of different spatial frequency resolution. a AO-off diffraction-limited image of a 100-nm-diameter bead with an aberration layer. Scale bar indicates 500 nm. **b** Same as **a**, but with AO (top), and the aberration correction map (bottom). Scale bar indicates 500 nm. **c**-**e** Same as **b**, but with the binning of the SLM pixels and thus varying the k-space resolution Δk to 2^4 (**c**), 2^6 (**d**), and 2^7 (**e**) SLM pixels, respectively. Scale bars indicate 500 nm. **f** Line profiles of the PSFs in **a**-**e** along the longest axis of AO-off image in **a**. **g** PSF FWHMs. FWHM of AO-off PSF is shown for reference. **h** Intensity change in bead images. The green line indicates decreasing intensity of 100-nm-diameter beads due to normal bleaching (without an aberration layer; without AO). Black vertical lines in **g** and **h** correspond to a deformable mirror with 25 actuators.

Aberration of a tissue-cleared whole zebrafish

As illustrated in Supplementary Figure 18, we discovered that there were still comparable levels of aberrations even after tissue clearing (see "Tissue clearing of zebrafish embryos" section in Methods). The aberrations measured at depths of 72 μm and 102 μm were 1.17-1.90 rad and 1.38-2.91 rad, respectively, in RMS wavefront distortion. This explicitly suggests that aberration correction remains a critical issue for SMLM imaging even after tissue clearing. Although tissue clearing can reduce photon loss by decreasing tissue scattering, it does not significantly reduce aberrations, most likely because wavefront distortion primarily originates from complex surface morphology.

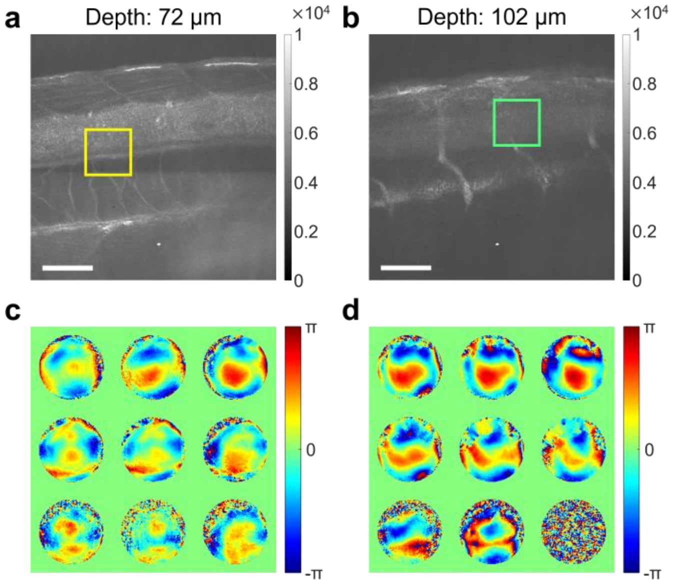

Supplementary Figure 18. Aberrations of a tissue-cleared whole zebrafish. a, **b** Confocal reflectance images of a tissue-cleared whole zebrafish at different depths. Due to clearing, the contrast of the reflectance image was significantly reduced. Scale bars indicate 50 μm. **c**, **d** Aberration maps measured at yellow and green boxes in **a** and **b**, respectively. Each aberration map corresponds to a subregion of ~10×10 μm^2.

Supplementary References

1. Burke, D., Patton, B., Huang, F., Bewersdorf, J. & Booth, M. J. Adaptive optics correction of specimen-induced aberrations in single-molecule switching microscopy. *Optica* **2**, 177 (2015).
2. Mlodzianoski, M. J. *et al.* Active PSF shaping and adaptive optics enable volumetric localization microscopy through brain sections. *Nat. Methods* **15**, 583–586 (2018).
3. Siemons, M. E., Hanemaaijer, N. A. K., Kole, M. H. P. & Kapitein, L. C. Robust adaptive optics for localization microscopy deep in complex tissue. *Nat. Commun.* **12**, 1–9 (2021).
4. Xu, F. *et al.* Three-dimensional nanoscopy of whole cells and tissues with in situ point spread function retrieval. *Nat. Methods* **17**, 531–540 (2020).
5. Nieuwenhuizen, R. P. J. *et al.* Measuring image resolution in optical nanoscopy. *Nat. Methods* **10**, 557–562 (2013).
6. Kim, M. *et al.* Label-free neuroimaging in vivo using synchronous angular scanning microscopy with single-scattering accumulation algorithm. *Nat. Commun.* **10**, 1–9 (2019).
7. Kang, S. *et al.* High-resolution adaptive optical imaging within thick scattering media using closed-loop accumulation of single scattering. *Nat. Commun.* **8**, (2017).
8. Kang, S. *et al.* Imaging deep within a scattering medium using collective accumulation of single-scattered waves. *Nat. Photonics* **9**, 253–258 (2015).
9. Kang, S. *et al.* High-resolution adaptive optical imaging within thick scattering media using closed-loop accumulation of single scattering. *Nat. Commun.* **8**, 2157 (2017).
10. Siemons, M. E., Hanemaaijer, N. A. K., Kole, M. H. P. & Kapitein, L. C. Robust adaptive optics for localization microscopy deep in complex tissue. *Nat. Commun.* **12**, 1–9 (2021).
11. Dempsey, G. T. *et al.* Photoswitching mechanism of cyanine dyes. *J. Am. Chem. Soc.* **131**, 18192–18193 (2009).
12. Gidi, Y. *et al.* Unifying Mechanism for Thiol-Induced Photoswitching and Photostability of Cyanine Dyes. *J. Am. Chem. Soc.* **142**, 12681–12689 (2020).
