# Supplementary material for: Label-free adaptive optics single-molecule localization microscopy for whole zebrafish
Source: Nat Commun. 2023 Jul 13;14:4185. doi: 10.1038/s41467-023-39896-2 (PMC10344925; doi:10.1038/s41467-023-39896-2)
Supplement: Supplementary file 3 — Reporting Summary [file 41467_2023_39896_MOESM3_ESM.pdf]

nature portfolio

Corresponding author(s): Wonshik Choi

Last updated by author(s): May 16, 2023

Reporting Summary

Nature Portfolio wishes to improve the reproducibility of the work that we publish. This form provides structure for consistency and transparency in reporting. For further information on Nature Portfolio policies, see our Editorial Policies and the Editorial Policy Checklist.

Statistics

For all statistical analyses, confirm that the following items are present in the figure legend, table legend, main text, or Methods section.

n/a	Confirmed	
☐	☒	The exact sample size (*n*) for each experimental group/condition, given as a discrete number and unit of measurement
☐	☒	A statement on whether measurements were taken from distinct samples or whether the same sample was measured repeatedly
☒	☐	The statistical test(s) used AND whether they are one- or two-sided<br>*Only common tests should be described solely by name; describe more complex techniques in the Methods section.*
☒	☐	A description of all covariates tested
☒	☐	A description of any assumptions or corrections, such as tests of normality and adjustment for multiple comparisons
☒	☐	A full description of the statistical parameters including central tendency (e.g. means) or other basic estimates (e.g. regression coefficient) AND variation (e.g. standard deviation) or associated estimates of uncertainty (e.g. confidence intervals)
☒	☐	For null hypothesis testing, the test statistic (e.g. *F*, *t*, *r*) with confidence intervals, effect sizes, degrees of freedom and *P* value noted<br>*Give P values as exact values whenever suitable.*
☒	☐	For Bayesian analysis, information on the choice of priors and Markov chain Monte Carlo settings
☒	☐	For hierarchical and complex designs, identification of the appropriate level for tests and full reporting of outcomes
☒	☐	Estimates of effect sizes (e.g. Cohen's *d*, Pearson's *r*), indicating how they were calculated

Our web collection on statistics for biologists contains articles on many of the points above.

Software and code

Policy information about availability of computer code

Data collection	Dedicated software (Camware 4.05, PCO): for controlling an sCMOS camera (pco.edge 4.2 m, PCO)<br>Dedicated software (NIS-Elements AR 5.30.03 64-Bit, Nikon): for controlling an EMCCD camera (DU-888U3-CS0-#BV, Andor)<br>Custom-developed code (MATLAB2020a, MathWorks): for controlling a two-axis galvo mirror (6210H, Cambridge Technology) and an SLM (X13138-06, Hamamatsu)<br>Dedicated software (Lucam Software v6.8.3, Teledyne Lumenera): for controlling a camera (LM135M, Teledyne Lumenera)<br>Dedicated software (APT Version 3.21.6, Thorlabs): for controlling a motorized stage (Z825B, Thorlabs)<br>Dedicated software (FWxC 5.0.0, Thorlabs): for controlling a motorized filter wheel (FW102C, Thorlabs)<br>REALM code rewritten in MATLAB language (MATLAB2020a, MathWorks): for REALM test (REALM authors' REALM code is not compatible with our SLM. So, we rewrote original REALM code in MATLAB language)
Data analysis	imageJ (imageJ version 1.53t, National Institutes of Health) with ThunderSTORM plugin (ThunderSTORM 1.3, GitHub): for ThunderSTORM analysis<br>Cutsom-developed codes (MATLAB2020a, MathWorks): for all other analyses except ThunderSTORM analysis

For manuscripts utilizing custom algorithms or software that are central to the research but not yet described in published literature, software must be made available to editors and reviewers. We strongly encourage code deposition in a community repository (e.g. GitHub). See the Nature Portfolio guidelines for submitting code & software for further information.

Data

Policy information about availability of data

All manuscripts must include a data availability statement. This statement should provide the following information, where applicable:

- Accession codes, unique identifiers, or web links for publicly available datasets
- A description of any restrictions on data availability
- For clinical datasets or third party data, please ensure that the statement adheres to our policy

The source data relevant to each figure or table is available from the corresponding authors upon reasonable request.

Human research participants

Policy information about studies involving human research participants and Sex and Gender in Research.

Reporting on sex and gender	N/A
Population characteristics	N/A
Recruitment	N/A
Ethics oversight	N/A

Note that full information on the approval of the study protocol must also be provided in the manuscript.

Field-specific reporting

Please select the one below that is the best fit for your research. If you are not sure, read the appropriate sections before making your selection.

☒ Life sciences ☐ Behavioural & social sciences ☐ Ecological, evolutionary & environmental sciences

For a reference copy of the document with all sections, see nature.com/documents/nr-reporting-summary-flat.pdf

Life sciences study design

All studies must disclose on these points even when the disclosure is negative.

Sample size	There was no statistical analysis which requires careful determining sample size. Instead, we imaged as many samples as possible due to heterogeneity of biological samples. Then, we chose the best images. They are main figures in the manuscript.
Data exclusions	Because of heterogeneity of biological samples, all kinds of samples were imaged many times. Then, only parts of them were selected. We excluded some trivial data such as accidentally defocused images, images not including interesting structures, etc.
Replication	Because of heterogeneity of biological samples, experiments were hardly reproduced. So, samples were imaged many times. Then, only parts of them were selected except trivial data. Then, among remained data, we chose the best images. They are main figures in the manuscript.
Randomization	There was no group allocation in this study.
Blinding	There was no group allocation in this study.

Reporting for specific materials, systems and methods

We require information from authors about some types of materials, experimental systems and methods used in many studies. Here, indicate whether each material, system or method listed is relevant to your study. If you are not sure if a list item applies to your research, read the appropriate section before selecting a response.

Materials & experimental systems

n/a	Involved in the study
☐	☒ Antibodies
☐	☒ Eukaryotic cell lines
☒	☐ Palaeontology and archaeology
☐	☒ Animals and other organisms
☒	☐ Clinical data
☒	☐ Dual use research of concern

Methods

n/a	Involved in the study
☒	☐ ChIP-seq
☒	☐ Flow cytometry
☒	☐ MRI-based neuroimaging

Antibodies

Antibodies used

Primary antibody for tubulin: ab6046, Abcam (diluted 1,000 times)
Secondary antibody for Alexa 647 immunolabeling: A-21245, Thermo Fisher (diluted 1,000 times)

Anti-GFP primary antibody for Alexa 647 immunolabeling: A31852, Thermo Fisher (diluted 2000 times)

Validation

Validation information is copied and pasted below with links.

ab6046, Abcam (https://www.abcam.com/products/primary-antibodies/beta-tubulin-antibody-loading-control-ab6046.html#lb)
- Western blot - Anti-beta Tubulin antibody - Loading Control (ab6046)
All lanes : Anti-beta Tubulin antibody - Loading Control (ab6046) at 1 µg/ml

Lane 1 : HeLa (Human epithelial cell line from cervix adenocarcinoma) whole cell lysate
Lane 2 : NIH3T3 (Mouse embryo fibroblast cell line) whole cell lysate
Lane 3 : PC12 (Rat adrenal gland pheochromocytoma cell line) whole cell lysate
Lane 4 : CHO/K1 (Chinese hamster ovary cell line) whole cell lysate

Lysates/proteins at 10 µg per lane.

Secondary
All lanes : Goat polyclonal to Rabbit IgG - H&L - Pre-Adsorbed (HRP) at 1/50000 dilution

Developed using the ECL technique.

Performed under reducing conditions.

Predicted band size: 50 kDa
Observed band size: 51 kDa

A31852, Thermo Fisher (https://www.thermofisher.com/antibody/product/GFP-Antibody-Polyclonal/A-31852)
- Antibody specificity was demonstrated by detection of different targets fused to GFP tag in transiently transfected lysates tested. Relative detection of GFP tag was observed across different proteins fused with GFP in H3-GFP (Lane 3-5) and p65-GFP (Lane 6). GFP-variant, YFP is also being detected in His-p65-YFP lysate (Lane 7), using Anti-GFP Polyclonal Antibody, Alexa Fluor 647 (Product # A-31852) in Western Blot.

Eukaryotic cell lines

Policy information about cell lines and Sex and Gender in Research

Cell line source(s)	COS-7 [AC28806, Korean Collection for Type Cultures (KCTC)]
Authentication	Cells were not authenticated
Mycoplasma contamination	Cell lines were not tested for mycoplasma contamination
Commonly misidentified lines (See ICLAC register)	No commonly misidentified cell lines were used in the study.

Animals and other research organisms

Policy information about studies involving animals; ARRIVE guidelines recommended for reporting animal research, and Sex and Gender in Research

Laboratory animals	Adult (over eight weeks old) Thy1-EGFP line M (Jackson Labs #007788) mice (raised at 20-22 °C and humidity of 50-55% with 12 h:12 h light/dark cycle)<br>Tg(bactin2::Arl13b-GFP) (#FRZCC1009) and Tg(claudinK:gal4vp16;uas:megfp) (#FRZCC1013) zebrafish embryos (3- to 5-dpf)
Wild animals	No wild animals were used in the study.

Reporting on sex	All observed structured in this study are observed regardless of sex. So, sex information has not been collected.
Field-collected samples	No field collected samples were used in the study.
Ethics oversight	All experimental procedures including animals were approved by the Committee of Animal Research Policy of Korea University [approval number KUIACUC-2019-24 (mouse) and KOREA-2021-0037 (zebrafish)].

Note that full information on the approval of the study protocol must also be provided in the manuscript.
