# Supplementary material for: Label-free adaptive optics single-molecule localization microscopy for whole zebrafish
Source: Nat Commun. 2023 Jul 13;14:4185. doi: 10.1038/s41467-023-39896-2 (PMC10344925; doi:10.1038/s41467-023-39896-2)
Supplement: Supplementary file 4 — Description of additional supplementary files [file 41467_2023_39896_MOESM4_ESM.pdf]

Description of additional supplementary files

Supplementary Movie 1: Comparison of blinking single-molecule PSF images of Figs. 1c-d. Movie of blinking single-molecule PSF images of Figs. 1c-d. Both AO-off and -on images were simultaneously recorded at each frame. Scale bar: 2.5 µm.
