# Supplementary figures and images for: Label-free adaptive optics single-molecule localization microscopy for whole zebrafish
Source: Nat Commun. 2023 Jul 13;14:4185. doi: 10.1038/s41467-023-39896-2 (PMC10344925; doi:10.1038/s41467-023-39896-2)

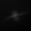

Supplement: Supplementary file 6 — Source data [file 41467_2023_39896_MOESM6_ESM.zip › Fig. 1/Fig. 1c Inset.tif]

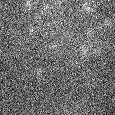

Supplement: Supplementary file 6 — Source data [file 41467_2023_39896_MOESM6_ESM.zip › Fig. 1/Fig. 1c.tif]

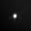

Supplement: Supplementary file 6 — Source data [file 41467_2023_39896_MOESM6_ESM.zip › Fig. 1/Fig. 1d Inset.tif]

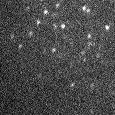

Supplement: Supplementary file 6 — Source data [file 41467_2023_39896_MOESM6_ESM.zip › Fig. 1/Fig. 1d.tif]

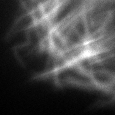

Supplement: Supplementary file 6 — Source data [file 41467_2023_39896_MOESM6_ESM.zip › Fig. 2/Fig. 2a.tif]

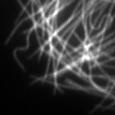

Supplement: Supplementary file 6 — Source data [file 41467_2023_39896_MOESM6_ESM.zip › Fig. 2/Fig. 2b.tif]

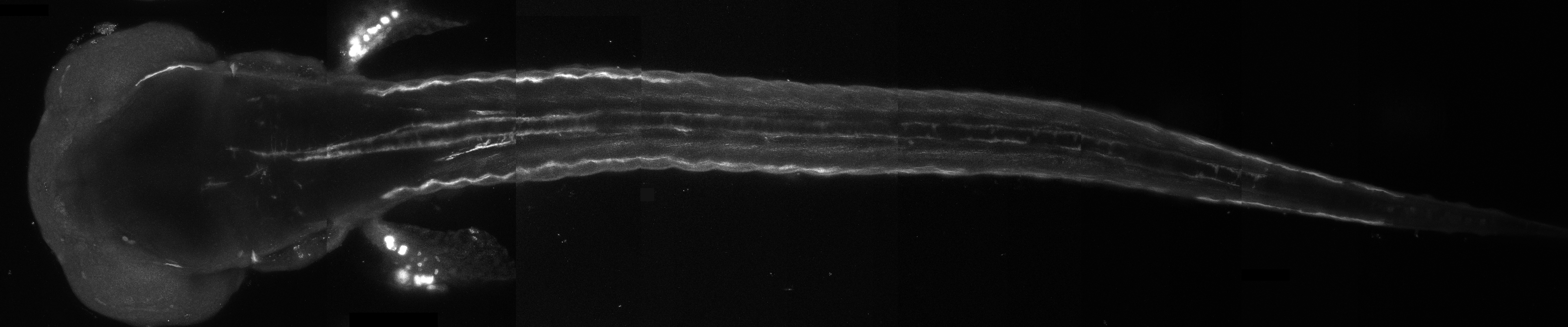

Supplement: Supplementary file 6 — Source data [file 41467_2023_39896_MOESM6_ESM.zip › Fig. 4/Fig. 4b Left.tif]

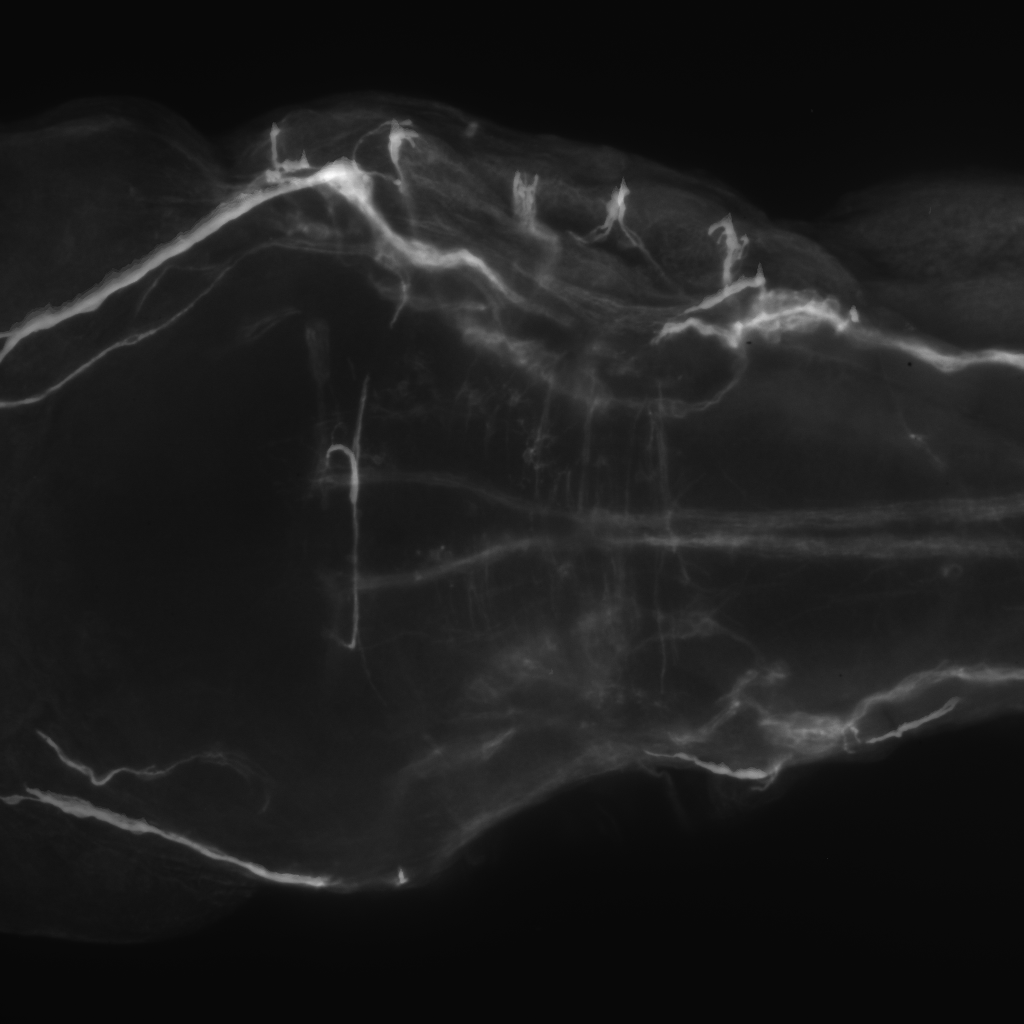

Supplement: Supplementary file 6 — Source data [file 41467_2023_39896_MOESM6_ESM.zip › Fig. 4/Fig. 4b Right.tif]

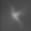

Supplement: Supplementary file 6 — Source data [file 41467_2023_39896_MOESM6_ESM.zip › Fig. 4/Fig. 4c Bottom Left.tif]

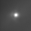

Supplement: Supplementary file 6 — Source data [file 41467_2023_39896_MOESM6_ESM.zip › Fig. 4/Fig. 4c Bottom Right.tif]

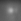

Supplement: Supplementary file 6 — Source data [file 41467_2023_39896_MOESM6_ESM.zip › Fig. 4/Fig. 4f Bottom Left.tif]

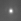

Supplement: Supplementary file 6 — Source data [file 41467_2023_39896_MOESM6_ESM.zip › Fig. 4/Fig. 4f Bottom Right.tif]

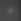

Supplement: Supplementary file 6 — Source data [file 41467_2023_39896_MOESM6_ESM.zip › Fig. 4/Fig. 4i Bottom Left.tif]

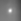

Supplement: Supplementary file 6 — Source data [file 41467_2023_39896_MOESM6_ESM.zip › Fig. 4/Fig. 4i Bottom Right.tif]

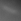

Supplement: Supplementary file 6 — Source data [file 41467_2023_39896_MOESM6_ESM.zip › Fig. 4/Fig. 4l Bottom Left.tif]

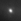

Supplement: Supplementary file 6 — Source data [file 41467_2023_39896_MOESM6_ESM.zip › Fig. 4/Fig. 4l Bottom Right.tif]

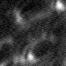

Supplement: Supplementary file 6 — Source data [file 41467_2023_39896_MOESM6_ESM.zip › Fig. S10/Fig. S10a Bottom.tif]

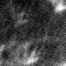

Supplement: Supplementary file 6 — Source data [file 41467_2023_39896_MOESM6_ESM.zip › Fig. S10/Fig. S10a Top.tif]

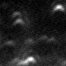

Supplement: Supplementary file 6 — Source data [file 41467_2023_39896_MOESM6_ESM.zip › Fig. S10/Fig. S10b Bottom.tif]

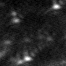

Supplement: Supplementary file 6 — Source data [file 41467_2023_39896_MOESM6_ESM.zip › Fig. S10/Fig. S10b Top.tif]

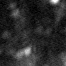

Supplement: Supplementary file 6 — Source data [file 41467_2023_39896_MOESM6_ESM.zip › Fig. S10/Fig. S10c Bottom.tif]

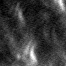

Supplement: Supplementary file 6 — Source data [file 41467_2023_39896_MOESM6_ESM.zip › Fig. S10/Fig. S10c Top.tif]

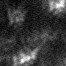

Supplement: Supplementary file 6 — Source data [file 41467_2023_39896_MOESM6_ESM.zip › Fig. S10/Fig. S10d Bottom.tif]

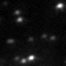

Supplement: Supplementary file 6 — Source data [file 41467_2023_39896_MOESM6_ESM.zip › Fig. S10/Fig. S10d Top.tif]

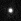

Supplement: Supplementary file 6 — Source data [file 41467_2023_39896_MOESM6_ESM.zip › Fig. S11/Fig. S11d Bottom.tif]

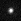

Supplement: Supplementary file 6 — Source data [file 41467_2023_39896_MOESM6_ESM.zip › Fig. S11/Fig. S11d Top.tif]

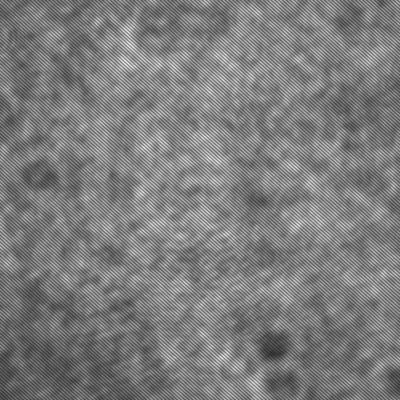

Supplement: Supplementary file 6 — Source data [file 41467_2023_39896_MOESM6_ESM.zip › Fig. S12/Fig. S12a Mirror Image.tif]

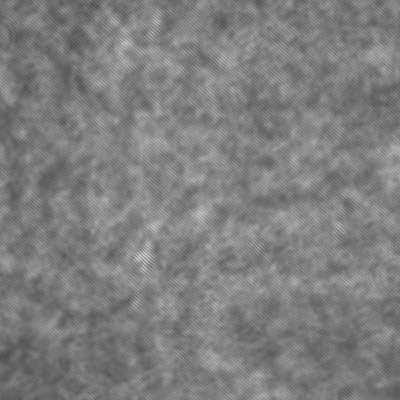

Supplement: Supplementary file 6 — Source data [file 41467_2023_39896_MOESM6_ESM.zip › Fig. S12/Fig. S12a Sample Image.tif]

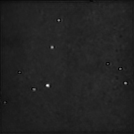

Supplement: Supplementary file 6 — Source data [file 41467_2023_39896_MOESM6_ESM.zip › Fig. S12/Fig. S12d Corrected.tif]

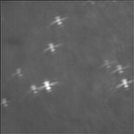

Supplement: Supplementary file 6 — Source data [file 41467_2023_39896_MOESM6_ESM.zip › Fig. S12/Fig. S12d Uncorrected.tif]

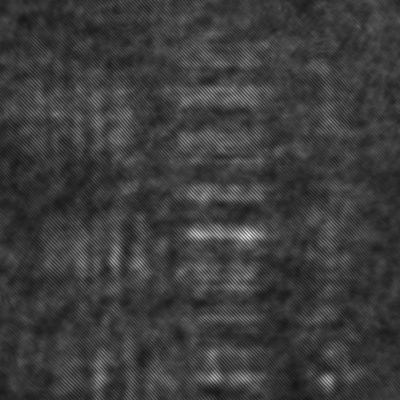

Supplement: Supplementary file 6 — Source data [file 41467_2023_39896_MOESM6_ESM.zip › Fig. S13/Fig. S13a.tif]

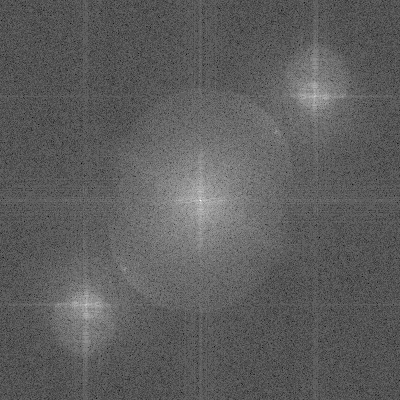

Supplement: Supplementary file 6 — Source data [file 41467_2023_39896_MOESM6_ESM.zip › Fig. S13/Fig. S13b.tif]

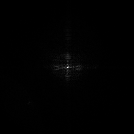

Supplement: Supplementary file 6 — Source data [file 41467_2023_39896_MOESM6_ESM.zip › Fig. S13/Fig. S13c.tif]

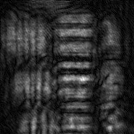

Supplement: Supplementary file 6 — Source data [file 41467_2023_39896_MOESM6_ESM.zip › Fig. S13/Fig. S13d.tif]

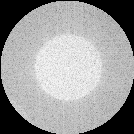

Supplement: Supplementary file 6 — Source data [file 41467_2023_39896_MOESM6_ESM.zip › Fig. S13/Fig. S13e.tif]

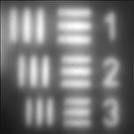

Supplement: Supplementary file 6 — Source data [file 41467_2023_39896_MOESM6_ESM.zip › Fig. S13/Fig. S13h.tif]

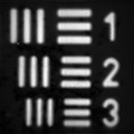

Supplement: Supplementary file 6 — Source data [file 41467_2023_39896_MOESM6_ESM.zip › Fig. S13/Fig. S13k.tif]

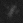

Supplement: Supplementary file 6 — Source data [file 41467_2023_39896_MOESM6_ESM.zip › Fig. S17/Fig. S17a.tif]

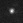

Supplement: Supplementary file 6 — Source data [file 41467_2023_39896_MOESM6_ESM.zip › Fig. S17/Fig. S17b.tif]

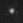

Supplement: Supplementary file 6 — Source data [file 41467_2023_39896_MOESM6_ESM.zip › Fig. S17/Fig. S17c.tif]

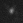

Supplement: Supplementary file 6 — Source data [file 41467_2023_39896_MOESM6_ESM.zip › Fig. S17/Fig. S17d.tif]

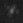

Supplement: Supplementary file 6 — Source data [file 41467_2023_39896_MOESM6_ESM.zip › Fig. S17/Fig. S17e.tif]

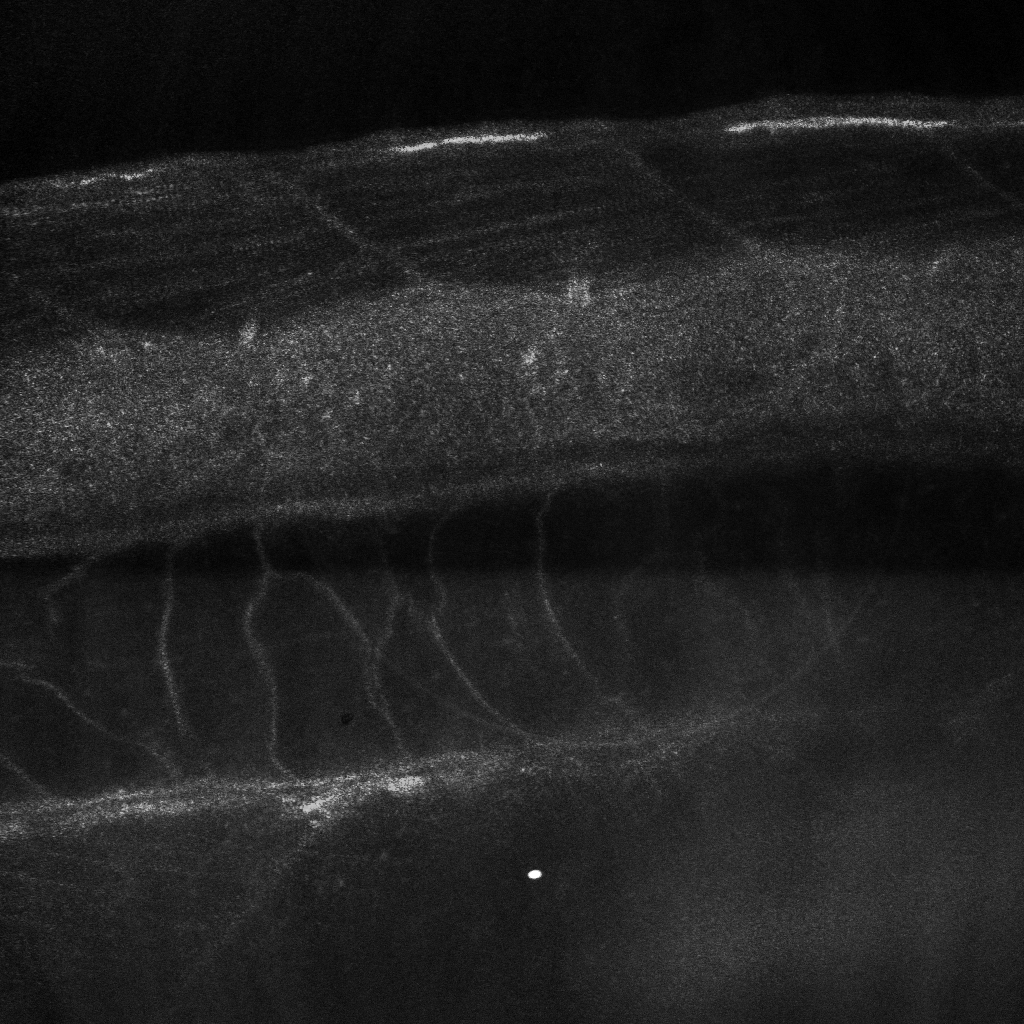

Supplement: Supplementary file 6 — Source data [file 41467_2023_39896_MOESM6_ESM.zip › Fig. S18/Fig. S18a.tif]

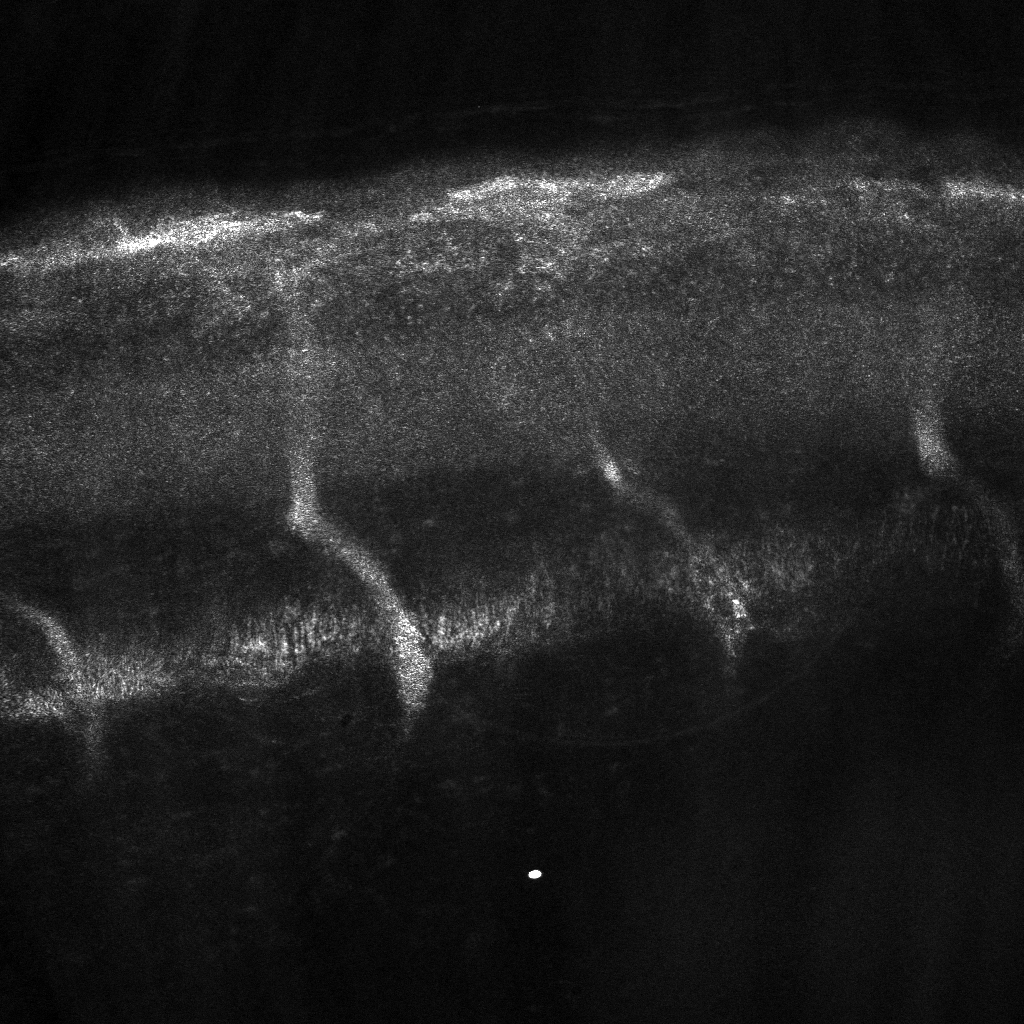

Supplement: Supplementary file 6 — Source data [file 41467_2023_39896_MOESM6_ESM.zip › Fig. S18/Fig. S18b.tif]

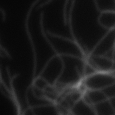

Supplement: Supplementary file 6 — Source data [file 41467_2023_39896_MOESM6_ESM.zip › Fig. S3/Fig. S3a.tif]

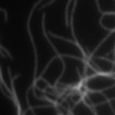

Supplement: Supplementary file 6 — Source data [file 41467_2023_39896_MOESM6_ESM.zip › Fig. S3/Fig. S3b.tif]

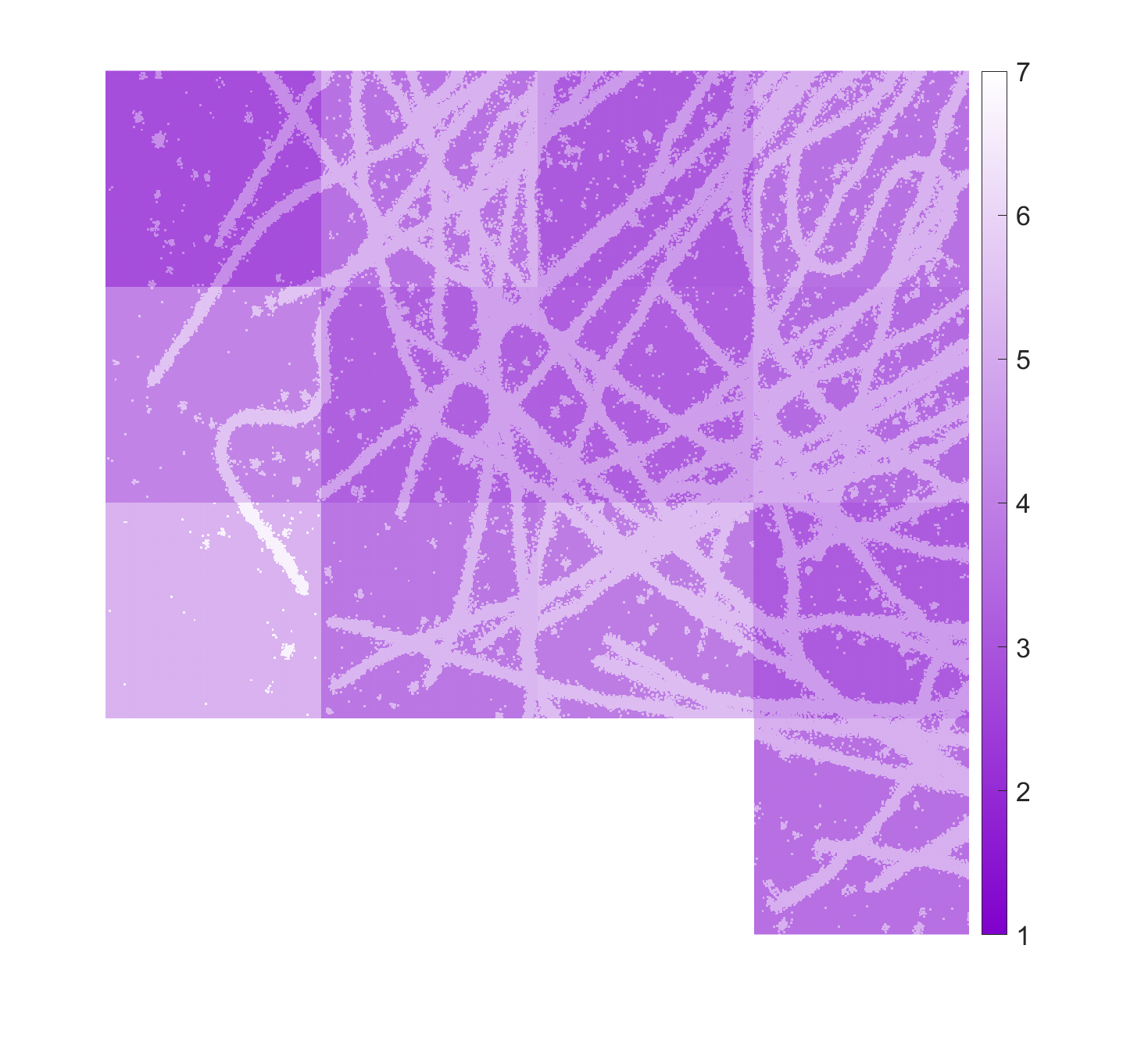

Supplement: Supplementary file 6 — Source data [file 41467_2023_39896_MOESM6_ESM.zip › Fig. S4/Fig. S4a.png]

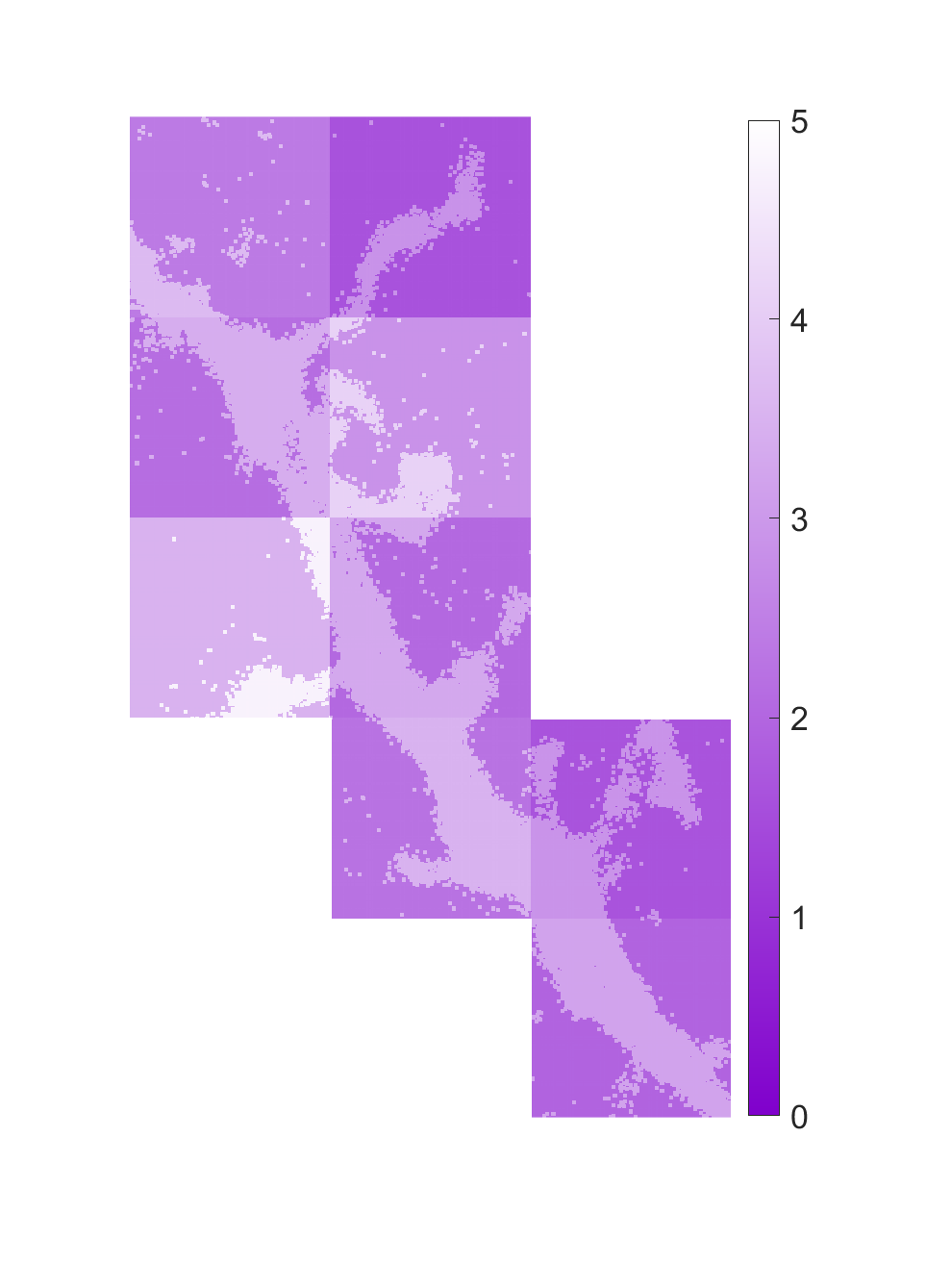

Supplement: Supplementary file 6 — Source data [file 41467_2023_39896_MOESM6_ESM.zip › Fig. S4/Fig. S4b.png]

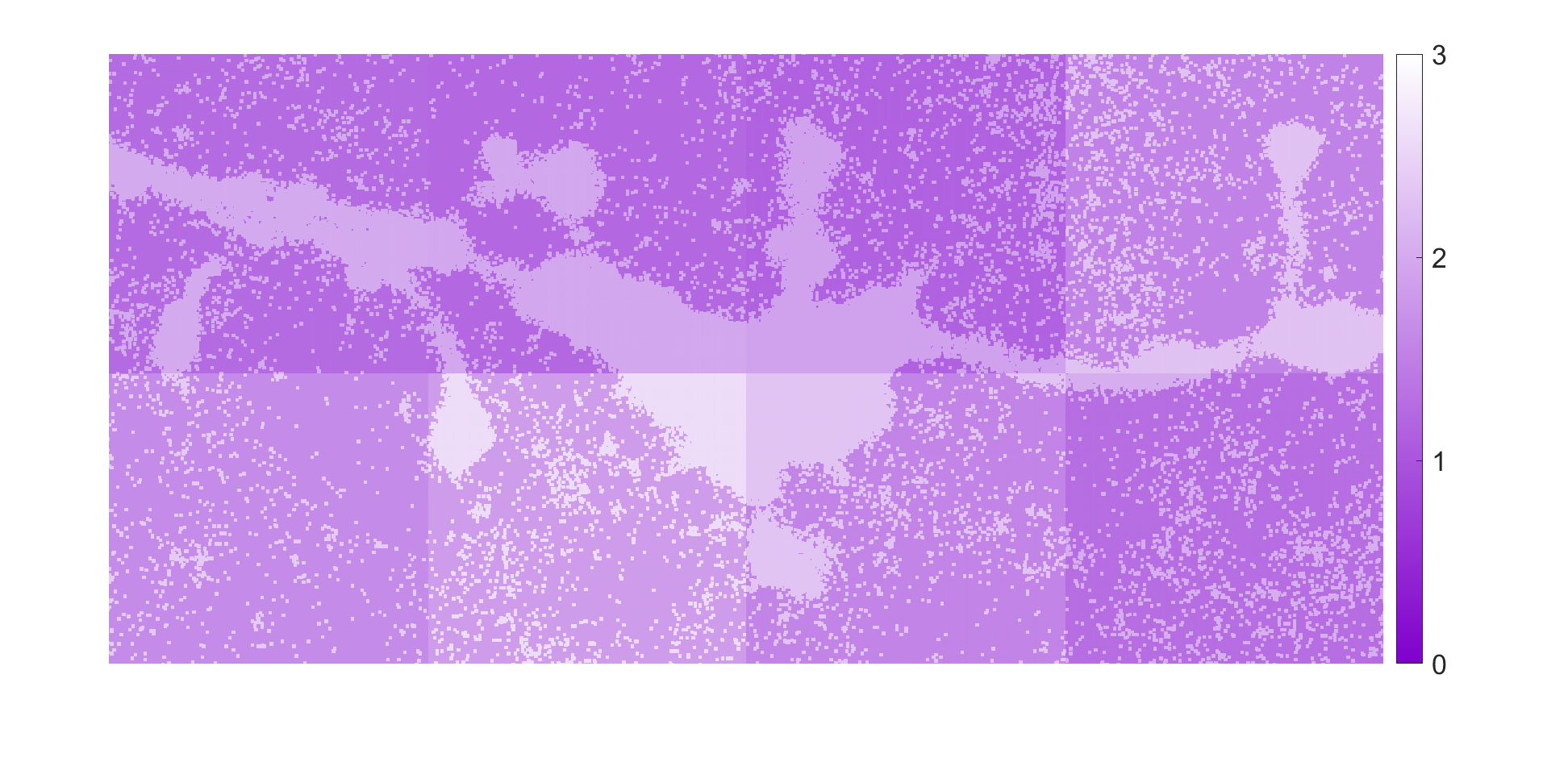

Supplement: Supplementary file 6 — Source data [file 41467_2023_39896_MOESM6_ESM.zip › Fig. S4/Fig. S4c.png]

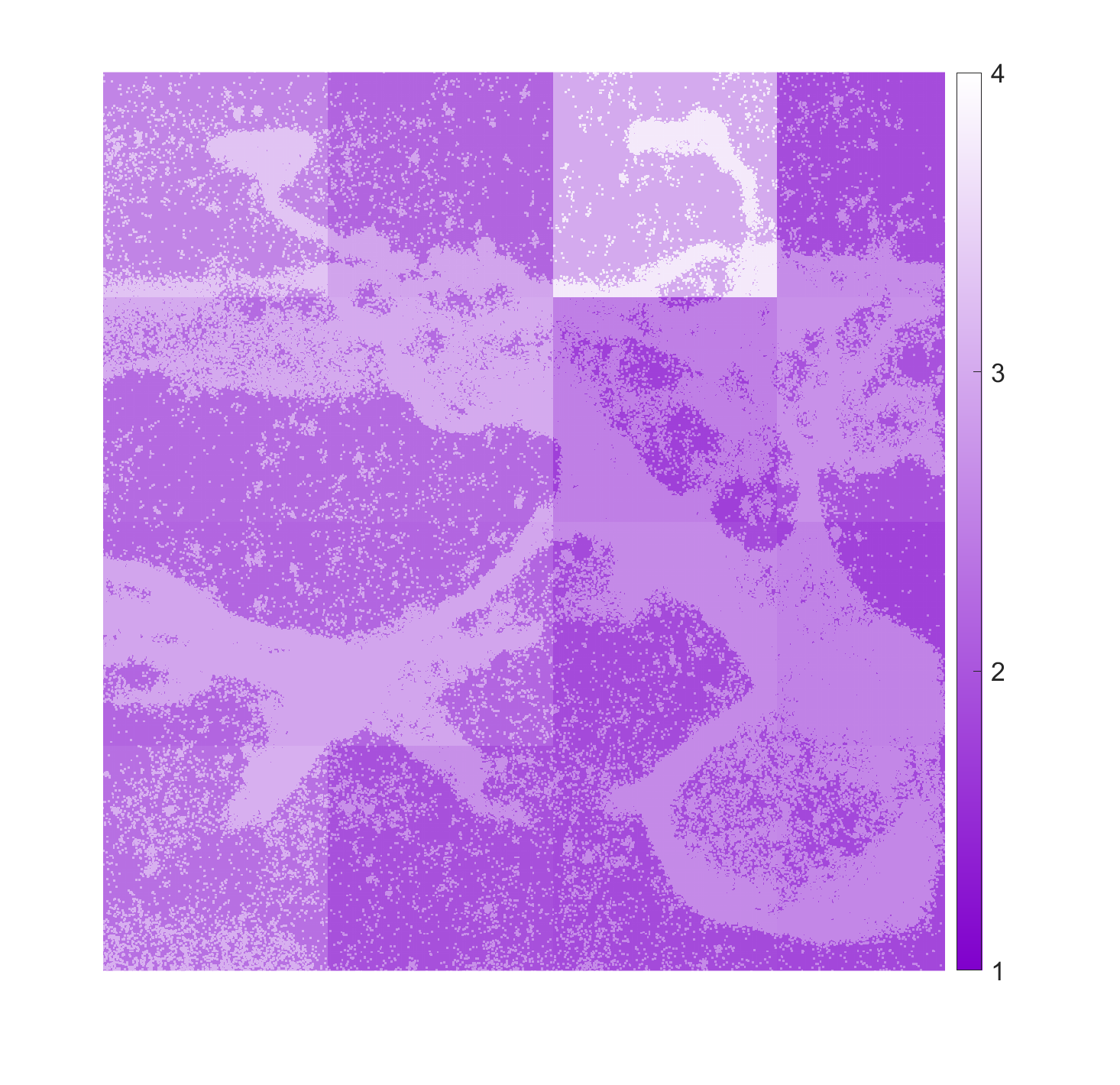

Supplement: Supplementary file 6 — Source data [file 41467_2023_39896_MOESM6_ESM.zip › Fig. S4/Fig. S4d.png]

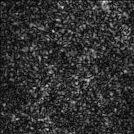

Supplement: Supplementary file 6 — Source data [file 41467_2023_39896_MOESM6_ESM.zip › Fig. S5/Fig. S5a Left.tif]

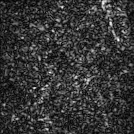

Supplement: Supplementary file 6 — Source data [file 41467_2023_39896_MOESM6_ESM.zip › Fig. S5/Fig. S5a Right.tif]

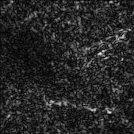

Supplement: Supplementary file 6 — Source data [file 41467_2023_39896_MOESM6_ESM.zip › Fig. S5/Fig. S5b Left.tif]

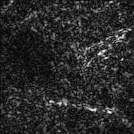

Supplement: Supplementary file 6 — Source data [file 41467_2023_39896_MOESM6_ESM.zip › Fig. S5/Fig. S5b Right.tif]

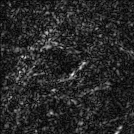

Supplement: Supplementary file 6 — Source data [file 41467_2023_39896_MOESM6_ESM.zip › Fig. S5/Fig. S5c Left.tif]

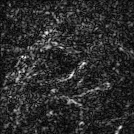

Supplement: Supplementary file 6 — Source data [file 41467_2023_39896_MOESM6_ESM.zip › Fig. S5/Fig. S5c Right.tif]

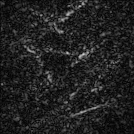

Supplement: Supplementary file 6 — Source data [file 41467_2023_39896_MOESM6_ESM.zip › Fig. S5/Fig. S5d Left.tif]

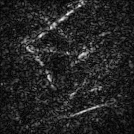

Supplement: Supplementary file 6 — Source data [file 41467_2023_39896_MOESM6_ESM.zip › Fig. S5/Fig. S5d Right.tif]

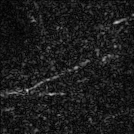

Supplement: Supplementary file 6 — Source data [file 41467_2023_39896_MOESM6_ESM.zip › Fig. S5/Fig. S5e Left.tif]

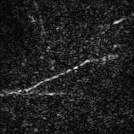

Supplement: Supplementary file 6 — Source data [file 41467_2023_39896_MOESM6_ESM.zip › Fig. S5/Fig. S5e Right.tif]

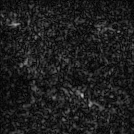

Supplement: Supplementary file 6 — Source data [file 41467_2023_39896_MOESM6_ESM.zip › Fig. S5/Fig. S5f Left.tif]

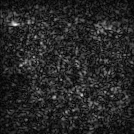

Supplement: Supplementary file 6 — Source data [file 41467_2023_39896_MOESM6_ESM.zip › Fig. S5/Fig. S5f Right.tif]

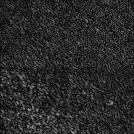

Supplement: Supplementary file 6 — Source data [file 41467_2023_39896_MOESM6_ESM.zip › Fig. S5/Fig. S5g Left.tif]

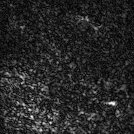

Supplement: Supplementary file 6 — Source data [file 41467_2023_39896_MOESM6_ESM.zip › Fig. S5/Fig. S5g Right.tif]

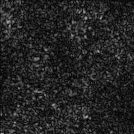

Supplement: Supplementary file 6 — Source data [file 41467_2023_39896_MOESM6_ESM.zip › Fig. S5/Fig. S5h Left.tif]

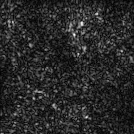

Supplement: Supplementary file 6 — Source data [file 41467_2023_39896_MOESM6_ESM.zip › Fig. S5/Fig. S5h Right.tif]
